# EnzRetro: Enzymatic Retrosynthetic Planning With Site‐Specific Reaction Edits Based on Sequence Generative Architecture

**DOI:** 10.1002/exp2.70129

**Published:** 2026-02-26

**Authors:** Yahui Cao, Haoshu Chen, Tao Zhang, Xin Zhao, Bingzhi Li, Xiangrui Zheng

**Affiliations:** ^1^ School of Electrical and Information Engineering Tianjin University Tianjin China; ^2^ Department of Biochemistry Yong Loo Lin School of Medicine National University of Singapore Singapore Singapore; ^3^ State Key Lab of Synthetic Biology Tianjin University Tianjin China; ^4^ School of Chemical Engineering and Technology Tianjin University Tianjin China

**Keywords:** biosynthesis, enzymatic retrosynthesis, enzyme identification, large language model

## Abstract

Enzymatic biosynthesis has become increasingly crucial in green chemistry and biosynthesis. However, current computational tools struggle to effectively integrate enzyme identification with pathway synthesis due to the specificity of enzymes and their complex interactions with substrates. Here, we propose EnzRetro, a novel framework for enzymatic retrosynthesis that provides an end‐to‐end solution bridging retrosynthesis planning with enzymatic engineering. The core innovative concept of EnzRetro is site‐specific reaction edits (SSREdits), a dynamic approach to representing structural transformations at specific enzyme active sites and forging a direct link between enzyme identification and reaction patterns. To enable the model to learn meaningful representations of SSREdits, we developed three pretraining tasks and then fine‐tuned two specialized models: (1) the SSREdits generation model for pathway synthesis, which translates the target product into a sequence of reaction edits, and (2) the EC generation model for enzyme identification, which focuses on precise transformation sites within SSREdits, enabling generalization across diverse reactions. Extensive experiments demonstrate the superior performance of EnzRetro, with a promising 56.1% and 97.7% Top‐1 accuracy on USPTO‐50k dataset for retrosynthesis and ECREACT dataset for enzyme identification, respectively. Finally, EnzRetro combines the two‐stage processes of retrosynthesis and enzyme identification into one‐pot learning, enhancing both computational efficiency and interpretability, and bridging the gap between pathway synthesis and enzyme identification. The accuracy of EnzRetro has been validated by three enzymatic pathways. We developed a web platform for multi‐step retrosynthesis planning that reconstructs multiple enzymatic pathways for putrescine biosynthesis with substantial diversity and significantly outperforms state‐of‐the‐art baselines.

## Graphical abstract

EnzRetro bridges retrosynthesis planning and enzymatic engineering through site‐specific reaction edits. This end‐to‐end framework integrates pathway synthesis and enzyme identification into a unified process, achieving superior accuracy and enabling the efficient reconstruction of diverse biosynthetic pathways.

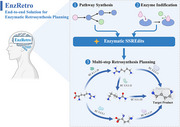

## 1 Introduction

Enzymatic biosynthesis has emerged as a cornerstone of modern green chemistry and industrial biotechnology, expanding access to complex molecules through substrate‐specific catalysis that streamlines purification and lowers environmental burden [1, 2, 3]. It provides a flexible and efficient approach to assembling enzymatic pathways for the production of high‐value compounds, such as pharmaceuticals [4, 5, 6], biofuels [7, 8, 9] and sustainable chemicals [10, 11, 12]. The design of efficient enzymatic pathways involves two critical stages: (1) pathway synthesis, where an optimal sequence of biochemical reactions is crafted to convert substrates into the desired product, and (2) enzyme identification, where enzymes with the appropriate specificity and catalytic efficiency are selected to execute each reaction step.

However, there poses a significant challenge of enzymatic retrosynthesis, whether in engineered cell factories or cell‐free systems: accurately modeling enzyme‐substrate specificity and effectively integrating it with pathway planning. Current computational methods often treat pathway synthesis and enzyme identification as separate tasks. This separation is further exacerbated by issues such as data scarcity, poor data quality, the complexity of enzyme specificity modeling, and the absence of a unified tool. These limitations not only reduce efficiency but also hinder the broader application of enzymatic retrosynthesis in metabolic engineering.

Retrosynthesis has gained increasing attention for developing synthetic pathways for target molecules, with single‐step retrosynthesis prediction focusing on breaking down the target molecule into simpler precursors, while multi‐step retrosynthesis planning involves exploring synthesis pathways over multiple reaction steps [13, 14, 15]. Existing AI‐based retrosynthesis methods achieve promising results and can be broadly categorized as selection‐based, generation‐based, and semi‐template‐based methods [16]. Selection‐based methods regard retrosynthesis prediction as a retrieval problem, focusing on selecting appropriate reactants or reaction templates from predefined sets based on prior knowledge [17, 18, 19, 20]. Due to the efficiency and interpretability of selecting and applying templates or reactants to generate feasible candidates, several works have been proposed to prioritize the candidates based on various algorithms [21, 22, 23]. However, the generalization and efficiency of selection‐based methods have been limited by the predefined templates or reactants, which makes them less flexible and less capable of discovering entirely new reaction pathways compared to more generative approaches. Generation‐based methods formulate retrosynthesis as a sequence‐to‐sequence (Seq2Seq) generation problem, generating molecular structures from scratch without any predefined sets or chemical knowledge [24, 25, 26, 27, 28, 29, 30, 31, 32, 33, 34, 35]. Most of the existing works employ the sequence representation of molecules, specifically the simplified molecular‐input line‐entry system (SMILES). While these methods have the potential to explore a wider range of possible reactions, they also present significant computational demands and a risk of generating invalid molecules. Semi‐template‐based methods involve a hybrid approach between selection‐based and generation‐based retrosynthesis [36, 37, 38, 39, 40, 41, 42]. They neither rely on predefined reaction templates nor directly convert the product into reactants in a single step. Instead, the semi‐template‐based methods involve two key stages: (1) identifying reaction centers, either atoms or bonds, to break down the target product into simpler intermediates or synthons, and (2) completing these synthons into reactants by leaving or attaching groups based on different models. In the first stage, several semi‐template‐based methods, such as G2Gs [36], GraphRetro [43], RetroXpert [37], G2Retro [42], RetroExplainer [44] and Graph2Edits [41], use graph neural networks (GNNs) or their variants to predict atom‐ and bond‐specific reaction centers. In the second stage, semi‐template‐based methods diverge in their approach to completing the reactants. Some methods are similar to selection‐based methods, where reactants are completed by selecting from predefined libraries. For example, GraphRetro [43] and RetroExplainer [44] select candidates from a library of leaving groups, while Graph2Edits [41] selects molecular edits and G2Retro [42] selects graph substructures. These methods are incapable of exploring a wider range of reactions and discovering more diverse retrosynthesis pathways. Other methods, like G2G [36] and RetroXpert [37], adopt graph or sequence generation models to generate novel reactants by synthesizing new structures based on the predicted synthons. However, these two‐stage methods often use two separate modules to complete the transformation, failing to capture important interactions between center identification and synthon completion in retrosynthesis. Besides, it poses a significant challenge to introduce ranking strategies to prioritize the generated or selected intermediates or synthons during the completion of the final reactants, making it challenging to ensure the chemical feasibility of retrosynthetic predictions. MEGAN [45] is of great significance as it marks the first attempt to use an end‐to‐end framework to convert product molecular graphs into the corresponding reactant graphs through a sequence of graph edits, but the performance was relatively low because it does not fully exploit the advantages of sequence generative models. Although existing AI‐driven retrosynthesis methods have achieved significant progress in generating synthetic pathways, they often fail to consider the availability of suitable enzymes to catalyze reactions effectively, creating a gap between theoretical pathway design and practical biocatalysis [46, 47, 48, 49].

To date, few studies have been attempted to incorporate enzyme identification into synthetic pathway prediction since the complexity of modeling enzyme specificity. BioNavi‐NP [50] was developed to predict the biosynthetic pathways, but it uses external tools to search for enzymes that catalyze the reactions. It separates pathway prediction from enzyme identification, requiring additional steps to link the predicted reactions with appropriate enzymes, which can limit the integration and efficiency of the overall biosynthesis prediction process. RetroBioCat [51] was the first method for predicting pathways by associating substrates with enzymes, but its reliance on predefined, expertly encoded reaction rules limits its scalability and ability to cover the full diversity of enzyme‐catalyzed reactions. Kreutter et al. [52] introduced a method by adopting the molecule transformer to predict the outcomes of enzymatic reactions for organic synthesis. This method trained the model on a dataset of 32,000 enzymatic reactions with the corresponding enzyme names. The complexity of the enzyme names leads to difficulties in accurately identifying and distinguishing between enzymes, and it only focuses on forward predictions, making it not applicable to retrosynthesis. Probst et al. [53] constructed the ECREACT dataset by incorporating the Enzyme Commission (EC) number into reaction SMILES instead of natural language name. They extended enzymatic reaction prediction to retrosynthesis by introducing a retro model that predicts both the reactants and the enzyme classes. While this method simplifies enzyme identification and extends enzymatic retrosynthesis, it may sacrifice precision and specificity due to the limitations of quantity and quality of the ECREACT dataset. In contrast, the USPTO dataset is a widely used benchmark dataset that includes millions of reactions and covers diverse reaction types, providing a strong foundation for pathway synthesis. The BEC‐Pred model [54], using a pretrain‐finetune architecture with pretraining on the USPTO dataset and fine‐tuning on ECREACT, applies reaction data to predict EC sub‐subclass labels, achieving state‐of‐the‐art performance with 91.6% accuracy. This method demonstrates the effectiveness of pretrain‐finetune architecture to extract enzyme information from rich reaction data for predicting enzyme functions, but it fails to address enzyme specificity and how enzymes selectively interact with structurally similar reactions. In particular, information about substrate modifications, product formation, and reaction transformations can serve as critical features for machine learning models. However, these data‐driven methods lack a detailed understanding of the deeper structural dynamics for enzyme‐reaction interactions, hindering model's generalizability and accuracy for synthesis pathways in metabolic engineering.

**FIGURE 1:**
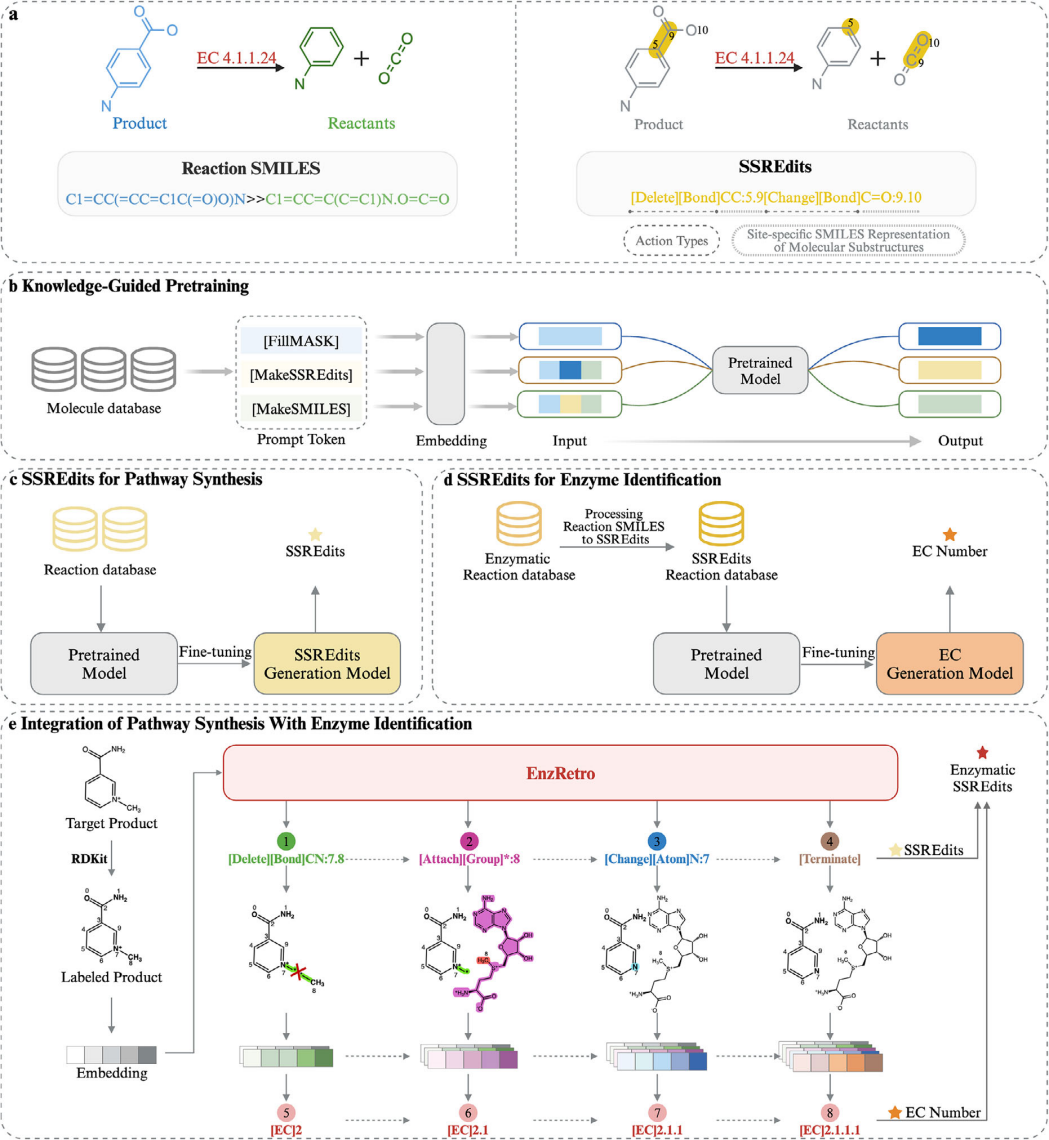
Overview of EnzRetro for enzymatic retrosynthesis planning. (a) The representation of reaction SMILES and SSREdits for a reaction catalyzed by the enzyme aminobenzoate decarboxylase (EC 4.1.1.24). There are two key components in SSREdits: action types and site‐specific SMILES representation of molecular substructures. (b) The pretraining strategy with three tasks for enhancing the model's understanding of SSREdits. (c,d) SSREdits for pathway synthesis and enzyme identification. The SSREdits generation model and EC generation model are fine‐tuned based on the pretrained model, and trained on reaction and enzymatic reaction databases, respectively. (e) EnzRetro, a unified tool that integrates pathway synthesis with enzyme identification. First, the target molecule is assigned corresponding IDs to each atom by RDKit tool, and then encoded them to generate SSREdits for specific atoms, bonds or groups. Next, the EC number of the enzyme is outputted sequentially by learning the comprehensive information present within SSREdits.

In this work Figure 1, EnzRetro is proposed for enzymatic retrosynthesis planning, providing an end‐to‐end solution that spans from enzyme selection to final production. Specifically, the approach formulates enzymatic retrosynthesis as a reasoning process, where products are transformed into reactants under the guidance of catalytic enzymes. By integrating reaction transformation and enzyme identification into one unified step, EnzRetro significantly enhances the efficiency, accuracy, and interpretability of enzymatic retrosynthesis planning.

1. We propose site‐specific reaction edits (SSREdits), a representative method for reaction transformation at specific enzyme active sites that contains comprehensive information on bond, atom, and group changes. The SSREdits include two key components: the action types and site‐specific SMILES representation of molecular substructures. The action types include eight defined operations, describing the stepwise transformations occurring during an enzymatic reaction. The site‐specific SMILES representations of molecular substructures are generated dynamically based on the input molecule provided to the model, as opposed to being derived from the training dataset or predefined by domain experts. Moreover, by focusing on detecting specific sites in the target molecule, our method eliminates ambiguity that arises when multiple possible sites are present for a given edit, ensuring precise and flexible retrosynthetic predictions. To enhance model's performance and generalization, we design three pretraining tasks targeting different aspects of SSREdits. The first task enables the model to learn the fundamental rules of SMILES for molecular substructures, while the latter two focus on capturing reaction transformation features and the relationship between reaction edits and SMILES representations.
2. For pathway synthesis, we develop a generative architecture for predicting SSREdits through fine‐tuning the pretrained model, introducing a new and distinct category of semi‐template‐based methods for retrosynthetic planning. This SSREdits generation model employs a Seq2Seq architecture to model single‐step retrosynthesis as a reaction reasoning process, where the target product is translated into a sequence of reaction edits at specific atomic sites. Our method streamlines the retrosynthesis process by completing it in a single step without additional generative modules or predefined substructure selection, offering a more accurate, flexible, and efficient solution for pathway planning. The proposed method achieves a Top‐1 exact‐match accuracy of 67.6% on USPTO‐50k for retro prediction and 90.4% on USPTO‐MIT for forward prediction, demonstrating its ability to effectively learn reaction transformations at specific atomic sites.
3. For enzyme identification, EnzRetro focuses on structural dynamics for enzyme‐reaction interactions. Inspired by the principle that identical reaction patterns correspond to the same enzyme, we observe in Table S1, Supporting information, that similar reaction edits consistently align with a shared EC number, highlighting the relationship between reaction transformations and enzyme specificity. Here, we introduce a generation model for EC number prediction based on the pretrained model. The EC number is a hierarchical numerical system for classifying enzymes based on the chemical reactions they catalyze. By using SSREdits as inputs to directly generate EC numbers sequences, the model learns to map the reaction transformations to enzyme classifications, thereby capturing structural patterns that define enzyme specificity.
4. Finally, we propose EnzRetro, an enzymatic SSREdits generation model that integrates reaction transformation and enzyme identification within one unified framework. This model translates the target product into enzymatic SSREdits, that is SSREdits annotated with the corresponding EC number. Compared to other semi‐template‐based methods that requires separate modules for center identification and synthon completion, or end‐to‐end frameworks that treat retrosynthesis as a Seq2Seq translation, EnzRetro offers distinct advantages: it performs true single‐step generation without relying on predefined substructure libraries or additional generative modules, while simultaneously bridging pathway synthesis with enzyme identification through a shared SSREdits representation. By focusing on active sites rather than the entire reaction, the model captures transformation‐specific features with high precision, enhances robustness to substrate variations, and ensures alignment between enzymatic function and reaction requirements. This targeted approach not only improves interpretability but also reduces input complexity and computational overhead, achieving superior efficiency without compromising performance. The proposed model is benchmarked on the ECREACT dataset, achieving promising results in comparison to other data‐driven methods. To validate the practicality and reliability of EnzRetro, we first performed sequential retrosynthesis to reconstruct three benchmark pathways, demonstrating high predictive accuracy. We then extended this into a multi‐objective retrosynthesis planning framework accessible via a web platform, which successfully recovered diverse biosynthetic pathways for putrescine, significantly outperforming baseline methods.

## 2 Results

### 2.1 SSREdits for Pathway Synthesis

#### 2.1.1 Performance Evaluation for Retrosynthesis and Forward Synthesis

To assess the effectiveness of the proposed SSREdits for pathway synthesis, we conducted experiments on two widely used benchmark datasets, USPTO‐50K and USPTO‐MIT, for retro and forward prediction, respectively. The performance of the SSREdits generation model was evaluated using Top‐k accuracy, with k set to 1, 3, 5, and 10.

Table 1 shows the performance of EnzRetro and other state‐of‐the‐art methods on the USPTO‐50K dataset for retro prediction. We compare the performance of EnzRetro with several selection‐based, generation‐based, and semi‐template‐based methods with or without reaction type information provided. EnzRetro outperforms all baselines besides the LocalRetro model when k equals 1, 3, and 5, with the exception of Top‐10 for known and unknown reaction types. Although our method did not achieve the highest level of accuracy, the Top‐10 accuracy is comparable to that of the optimal model (LocalRetro), with a difference of only 0.7% and 0.3% under reaction classes known and unknown, respectively. In order to facilitate a more precise comparison, EnzRetro demonstrated superior performance for semi‐template‐based methods across all metrics. It is more accurate than Graph2Edits and the MEGAN model, with a margin of 3.4% and 8% in Top‐1 accuracy when the reaction type is unknown.

**TABLE 1:** Performance of our EnzRetro and the state‐of‐the‐art methods on USPTO‐50K benchmarks for retrosynthesis prediction.

Model	Top‐k accuracy (%)							
	Reaction class unknown				Reaction class known			
	k = 1	3	5	10	1	3	5	10
**Selection‐based methods**								
Retrosmi [17]	37.3	54.7	63.3	74.1	52.9	73.8	81.2	88.1
Nerualsym [18]	44.4	65.3	72.4	78.9	55.3	76	81.4	85.1
GLN [19]	52.5	69	75.6	83.7	64.2	79.1	85.2	90
LocalRetro [20]	53.4	77.5	85.9	**92.4**	63.9	86.8	92.4	**96.3**
**Generation‐based methods**								
SCROP [26]	43.7	60	65.2	68.7	59	74.8	78.1	81.1
Aug. Transformer [29]	53.2	—	80.5	85.2	—	—	—	—
GTA [31]	51.1	67.6	74.8	81.6	—	—	—	—
Graph2SMILES [33]	52.9	66.5	70	72.9	—	—	—	—
Dual‐TF [32]	53.6	70.7	74.6	77	65.7	81.9	84.7	85.9
EditRetro [35]	52.2	67.1	71.6	74.2	60.8	80.6	86	90.3
**Semi‐template‐based methods**								
G2G [36]	48.9	67.6	72.5	75.5	61	81.3	86	88.7
RetroXpert [37]	50.4	61.1	62.3	63.4	62.1	75.8	78.5	80.9
RetroPrime [39]	51.4	70.8	74	76.1	64.8	81.6	85	86.9
MEGAN [40]	48.1	70.7	78.4	86.1	60.7	82	87.5	91.6
GraphRetro [43]	53.7	68.3	72.2	75.5	63.9	81.5	85.2	88.1
Graph2Edits [41]	52.7	77.2	85.3	91	65.7	87.3	92	95.3
G2Retro [42]	54.1	74.1	81.2	86.7	63.6	83.6	88.4	91.5
**EnzRetro (ours)**	**56.1**	**78.6**	**86.4**	91.7	**67.6**	**87.9**	**92.2**	95.6

*Note*: The results are taken from Graph2Edits [41].

To validate the effectiveness of EnzRetro for forward synthesis, in Table 2, we compare it with current state‐of‐the‐art methods on the USPTO‐MIT dataset in Top‐k predictions. EnzRetro achieved the best performance with a large margin in all metrics (Top‐1, ‐3, ‐5, and ‐10 accuracy). More specifically, EnzRetro outperformed the molecular transformer by 1%, 4.6%, 4.4%, and 4.2% in Top‐1, Top‐3, Top‐5, and Top‐10 accuracy on the mixed dataset, respectively; similar results can be seen on the separated dataset. These results indicate that EnzRetro is not only effective for retrosynthesis but also provides robust predictions applicable to forward synthesis, highlighting its versatility in addressing diverse synthetic challenges.

**TABLE 2:** Performance of our EnzRetro and the state‐of‐the‐art methods on USPTO‐MIT benchmarks for forward synthesis prediction.

Model	Top‐k accuracy (%)							
	Mixed				Separated			
	k = 1	3	5	10	1	3	5	10
Seq2Seq [55]	—	—	—	—	80.3	84.7	97.5	—
WLDN [56]	—	—	—	—	80.6	90.5	93.4	—
Molecular Transformer [57]	88.6	92.4	94.2	—	88.8	92.6	94.4	—
T5Chem [58]	88.9	92.9	95.2	—	90.4	94.2	96.4	—
MEGAN [45]	86.3	92.4	94.0	95.4	89.3	94.4	95.6	96.7
**EnzRetro (ours)**	**89.7**	**97.7**	**98.6**	**99.1**	**90.4**	**97.7**	**98.4**	**99.1**

*Note*: The results are taken from MEGAN [45] and T5Chem [58].

#### 2.1.2 SSREdits Generation Model for Enzymatic Reactions

**FIGURE 2:**
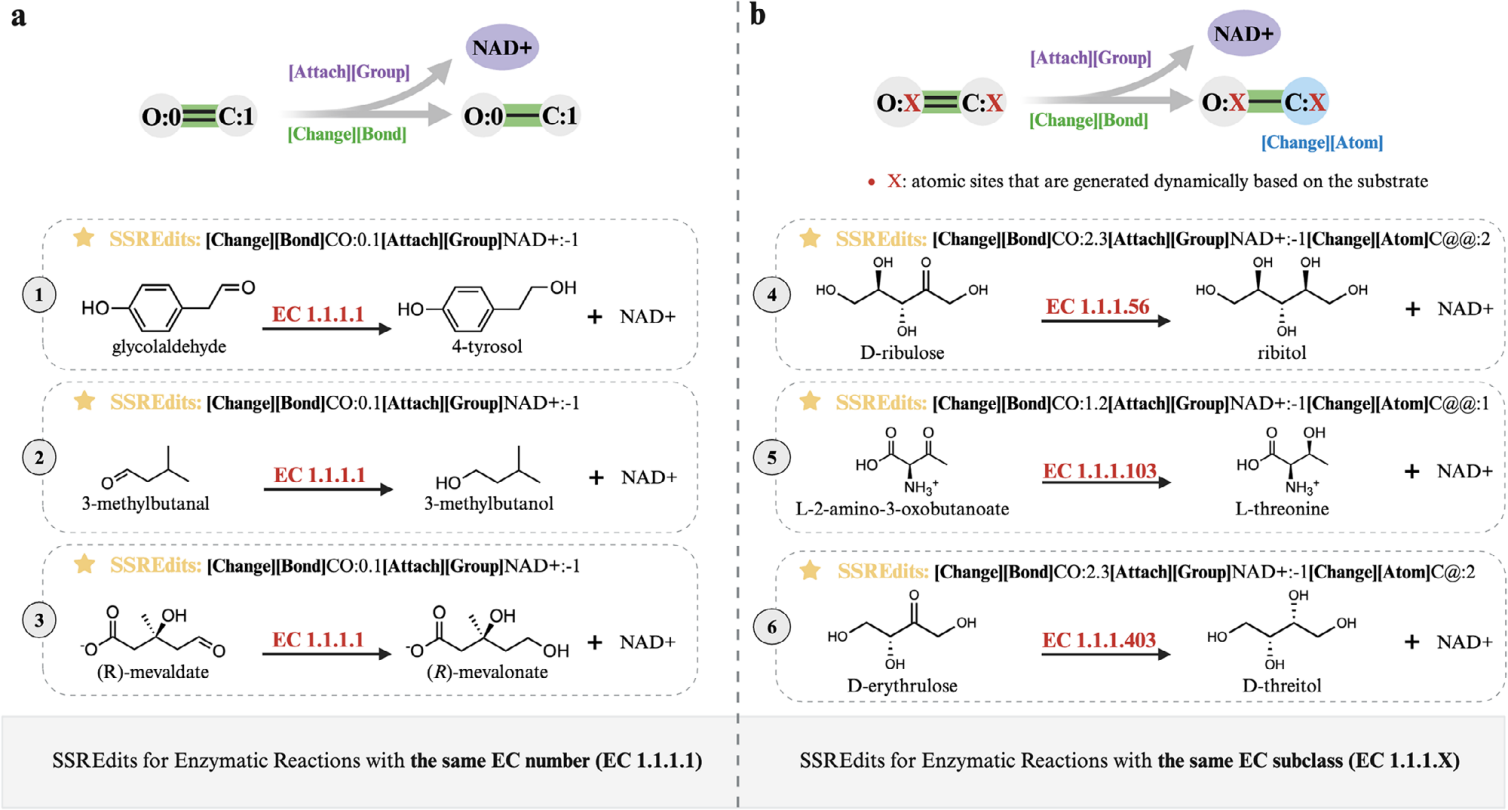
The SSREdits for enzymatic reactions with the identical EC number or within the identical EC subclass. (a) The SSREdits for enzymatic reactions with the same EC number (EC 1.1.1.1). (b) The SSREdits for enzymatic reactions within the same EC subclass (EC 1.1.1.X).

To further validate the relationship between reaction transformation and enzyme identification, we extend the SSREdits generation model to predict enzymatic reactions, and the predicted SSREdits for enzymatic reactions with the identical EC number or within the identical EC subclass are illustrated in Figure 2.

As shown in Figure 2a, enzymatic reactions catalyzed by enzymes with the same EC number generate identical SSREdits, reflecting the same reaction mechanism, irrespective of substrate variations. This alignment is exemplified by the enzyme's two‐step catalytic process: (1) reduction of the carbonyl double bond and (2) attachment of the NAD+ for generating a chiral center in substrates. Since EC numbers are assigned based on biochemical reactions catalyzed by enzymes, the consistency of SSREdits for the identical EC number across different products highlights that the model effectively encodes the reaction transformation logic intrinsic to that enzyme.

Figure 2b shows the generalization of SSREdits across EC subclasses, where reactions catalyzed by enzymes within the same EC subclass produce SSREdits that are structurally similar but differ in atomic site specificity. This observation reflects the fact that enzymes in the same EC subclass (e.g., oxidoreductases acting on CH‐OH groups) share a general reaction mechanism but may act on different substrates or in slightly different ways. For instance, as shown in Figure 2b, ribitol 2‐dehydrogenase(EC 1.1.1.56) catalyzes the retrosynthetic reduction of D‐ribulose to ribitol, L‐threonine dehydrogenase (EC 1.1.1.103) facilitates the retrosynthetic reduction of L‐2‐amino‐3‐oxobutanoate to L‐threonine, and D‐threitol dehydrogenase(EC 1.1.1.403) enables the retrosynthetic reduction of D‐erythrulose to D‐threitol. Both SSREdits involve three action steps: change bond, attach group and change atom, which reflect the mechanistic framework observed in Figure 2a. However, they target different atomic sites and induce distinct chirality depending on the substrate structure. Specifically, reaction 4 reduces D‐ribulose at the C2 position to produce ribitol, while reaction 6 reduces L‐2‐amino‐3‐oxobutanoate at the C1 position to yield L‐threonine. Furthermore, the chirality induced in reactions 4 and 6 is denoted by @@ in SMILES notation, whereas in reaction 5, it is represented by @. The site specificity reflects the substrate‐dependent nature of enzymatic catalysis, where the enzyme's active site geometry dictates the precise location of molecular modifications. EnzRetro successfully captures the transformation patterns while flexibly adjusting the details of atomic sites to fit the specific products.

The results demonstrate consistency in SSREdits for identical EC numbers and generalization in SSREdits across EC subclass, critical for bridging reaction transformation and enzyme identification.

### 2.2 SSREdits for Enzyme Identification

Inspired by the consistency and generalization of SSREdits, the EC generation model is introduced to identify corresponding enzymes for enzymatic reactions. We conducted experiments to predict EC numbers for enzymatic reactions on the ECREACT dataset, which is available at four levels (EC X.‐.‐.‐, EC X.X.‐.‐, EC X.X.X.‐ and EC X.X.X.X). To further evaluate the model's generalization capability, we also assessed its performance on the BioCyc database [59].

#### 2.2.1 Performance Evaluation on ECREACT

**FIGURE 3:**
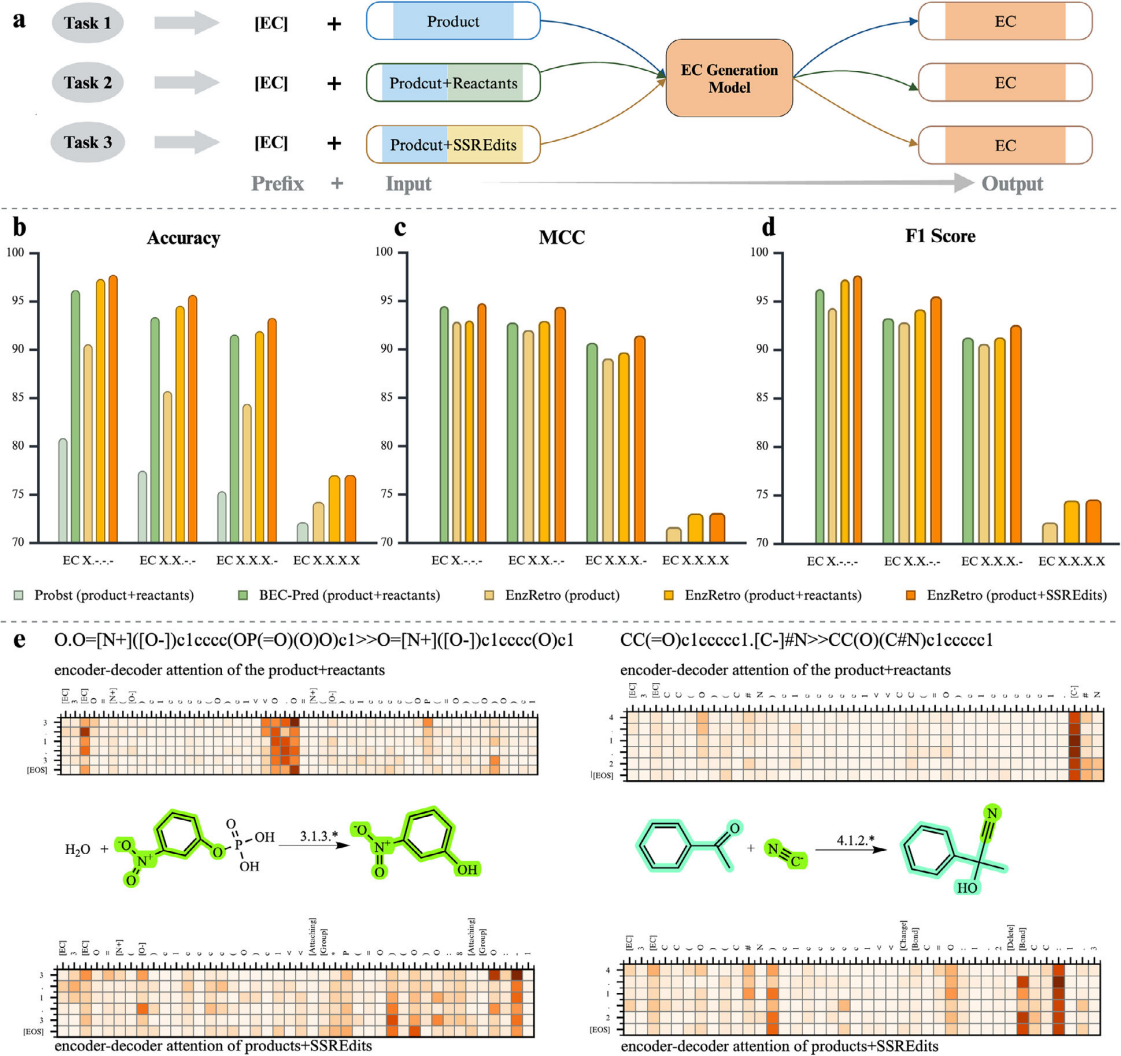
Framework and performance comparison of SSREdits for enzyme identification on the ECREACT dataset. (a) Three tasks are designed to validate EnzRetro's performance of the EC number predictions. These tasks predict EC numbers using the product, Product+Reactants and Product+SSREdits as input, respectively. (b–d) Performance of EnzRetro with three tasks and state‐of‐the‐art methods at different EC levels. (e) Attention weights interpretation for the EC generation model on EC X.X.X.‐. The horizontal axis represents the tokens of Product+Reactants (above) or Product+SSREdits (below), and the vertical axis refers to the output of the EC number. The darker the token, the more attention a specific token has received in that particular layer or output step. The colouring of the chemical reaction visually maps the transitions from reactants to the product in the ground truth.

Figure 3 demonstrates the framework and performance of the EC generation model on the ECREACT dataset. Figure 3a shows three prediction tasks designed to validate EnzRetro's ability to link SSREdits with EC classification. Task 1 predicts EC numbers using only the product's SMILES as input. Task 2 and Task 3 assess how reaction context enhances EC prediction accuracy using two input configurations: Product+Reactants and Product+SSREdits. Unlike previously developed machine learning algorithms that frame EC number prediction tasks as a multilabel classification problem, EnzRetro employed an autoregressive model to learn the representations of products, reactants and reaction edits in tasks.

Figure 3b–d compares the accuracy, Matthews correlation coefficient (MCC) and F1 score on the ECREACT dataset of EnzRetro and other state‐of‐the‐art methods, respectively. From EC X.‐.‐.‐ to EC X.X.X.‐, EnzRetro consistently outperformed other methods, demonstrating its superior performance in predicting EC numbers. These results are quite revealing in several ways. First, the advantage of pretrain‐finetune architecture. EnzRetro and BEC‐Pred [54] performed better than Probst's method [53] across all metrics, since these two methods apply pretrain‐finetune architecture to pretrain on the large‐scaled reaction dataset and fine‐tune on the ECREACT dataset. Second, the effectiveness of the proposed pretraining strategy. Using the Product+Reactants as input in the same format, EnzRetro still surpassed the BEC‐Pred model, highlighting its strength in identifying the correct EC number as the highest‐ranked prediction. These experimental results demonstrate that the pretraining strategy is capable of capturing comprehensive information about molecular structure and reaction transformation, providing more accurate EC number predictions at various hierarchical levels. Finally, the superiority of SSREdits. To investigate why EnzRetro performs better than other models on understudied EC numbers, we further conduct more in‐depth studies of our proposed EnzRetro using two different input combinations: Product+Reactants and Product+SSREdits. The results demonstrate that EnzRetro with Product+SSREdits exhibits superior performance compared to the one using Product+Reactants, achieving higher accuracy across all EC levels. This slight but consistent advantage indicates that incorporating SSREdits as input enables the model to better capture the functional and mechanistic details of enzymatic reactions, leading to more accurate predictions. In contrast, while reactants provide essential context for the reaction, they may lack the explicit representation of functional changes captured by reaction edits, resulting in slightly lower performance. This finding highlights the importance of representing reaction mechanisms explicitly in enzyme function prediction tasks. In Figure 3b, the results demonstrate a clear trend of decreasing accuracy as the specificity of the EC level increases. There is a gradual decline from EC X.‐.‐.‐ to EC X.X.X.‐, followed by a sharp drop from EC X.X.X.‐ to EC X.X.X.X, which is attributed to the significant increase in the number of unique entries from 284 to 5618. At EC X.X.X.‐, the sub‐subclass classification focuses on specific details about the reaction, offering a balance between specificity and complexity. Besides, Probst et al. [53] point out that EC X.X.X.‐ is the one with the richest amount of statistically significant information.

To examine whether EnzRetro's prediction accuracy differs across enzyme functional classes, we further evaluated EnzRetro's performance at each hierarchical level. The detailed results are provided in Figure S3, Supporting Information. At EC X.‐.‐.‐, EnzRetro achieved >90% Top‐1 accuracy for oxidoreductases (EC 1), transferases (EC 2), hydrolases (EC 3), lyases (EC 4), and ligases (EC 6), with transferases performing best (99.7% Top‐1). Isomerases (EC 5) showed moderately lower accuracy (79.6% Top‐1), likely due to their more complex rearrangement mechanisms and fewer training examples. As specificity increased (EC X.X.X.X), accuracy declined across all classes, with transferases remaining the most robust (92.8% Top‐1) and isomerases exhibiting the largest drop (24.5% Top‐1). Translocases (EC 7) was excluded from this analysis due to insufficient data in the ECREACT dataset. These variations reflect differences in data availability, mechanistic complexity, and the specificity of the reaction transformations captured by SSREdits. Overall, EnzRetro generalizes well across the major enzyme classes, with performance aligning with the representativeness and mechanistic clarity of each class in the training data.

#### 2.2.2 Visualization of Attention Weights

As illustrated in Figure 3e, the attention mechanisms in EnzRetro do provide interpretable insights into enzyme–reaction relationships. These two examples were generated by the model trained on the ECREACT dataset at EC X.X.X.‐. For the model with Product+Reactants as inputs, the model demonstrates a greater emphasis on a few specific tokens, indicating that it relies heavily on certain key elements of the input to make predictions. This concentrated attention suggests that the model prioritizes specific reactants or product features it deems critical for identifying the EC number. However, this narrow focus could limit the model's ability to generalize across diverse reaction contexts. For the model with Product+SSREdits, the encoder–decoder attention weights are more broadly and evenly distributed across the input tokens. Notably, the model pays more attention to the SSREdits segment than to the reactants. By focusing on these SSREdits, the attention mechanism highlights which structural changes are most relevant to enzyme function. It demonstrates that EnzRetro not only predicts EC numbers accurately but also reveals the underlying reaction features that determine enzyme selectivity. The balanced attention across inputs enhances the model's ability to extract relevant information from both the product and SSREdits.

#### 2.2.3 Generalization Evaluation on BioCyc Database

To address potential concerns regarding overfitting from the pretrain‐finetune strategy (from USPTO to ECREACT) and to evaluate generalization to entirely new reaction types, we conducted an additional validation experiment. The fine‐tuned EC generation model was applied to the BioCyc database, which is an independent, large‐scale repository of enzymatic reactions that was not used during any stage of training (pretraining or fine‐tuning). BioCyc encompasses a broader diversity of organisms and pathways compared to ECREACT, providing a rigorous test for model generalization.

The model's performance on BioCyc for EC number prediction across the four main enzyme classes is summarized in Figure S4, Supporting Information. The results demonstrate robust generalization, particularly for EC X.‐.‐.‐ to EC X.X.X.‐ reactions. For instance, the model achieved a Top‐3 accuracy of 95.5%, 88.1%, and 81.0% for EC X.‐.‐.‐, EC X.X.‐.‐ and EC X.X.X.‐, respectively. As expected, performance was lower for EC X.X.X.X reactions (55.5% Top‐3 accuracy), which is consistent with their broader mechanistic diversity and relatively lower representation in the training corpus, a known challenge in enzymatic function prediction. These results confirm that the pretrain‐finetune strategy did not lead to overfitting on ECREACT and that EnzRetro retains a strong capacity to predict EC numbers for novel and unrelated enzymatic transformations.

### 2.3 EnzRetro Integrates Pathway Synthesis With Enzyme Identification

#### 2.3.1 Performance Evaluation for Enzymatic SSREdits Prediction

**FIGURE 4:**
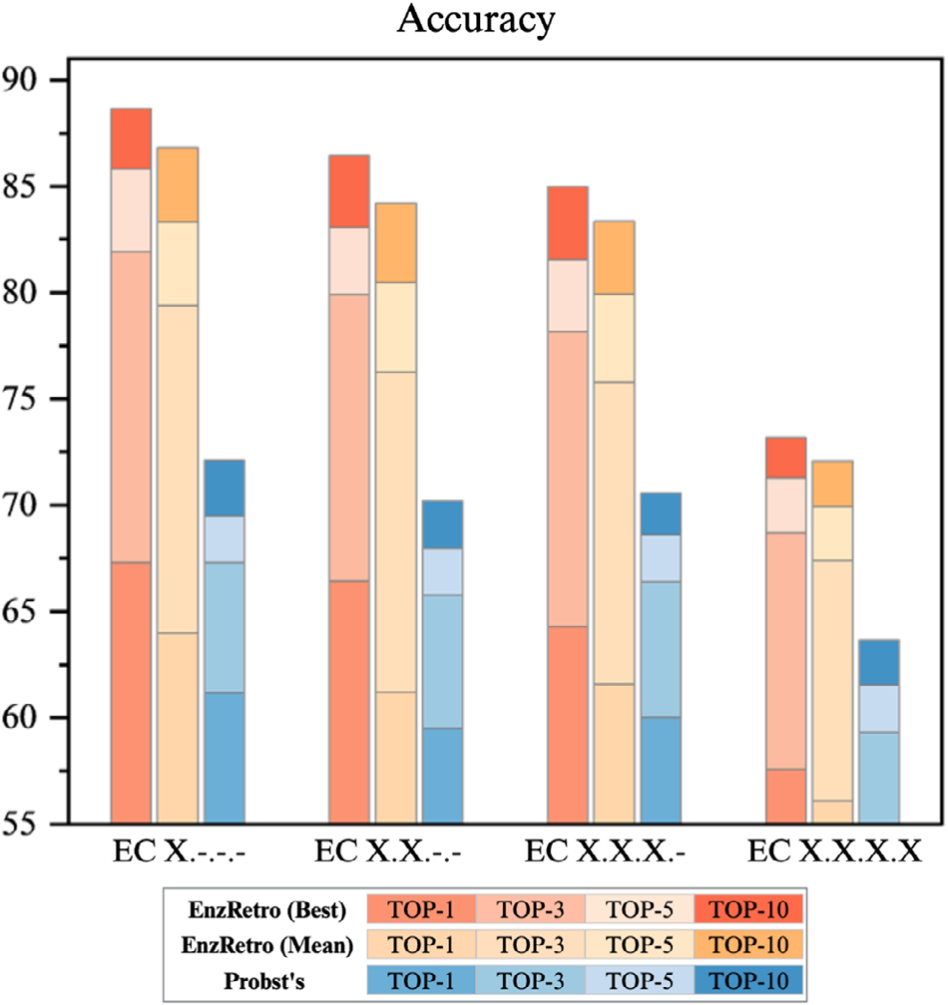
The performance evaluation of EnzRetro for enzymatic SSREdits prediction across different levels of EC number, in terms of Top‐1, Top‐3, Top‐5, and Top‐10. Top‐k represents the exact match accuracy in identifying the ground‐truth result within the model's Top‐k predictions. The probabilities associated with each SSREdit step are the model's token‐wise confidence scores generated autoregressively.

Enzymatic SSREdits prediction is a one‐pot prediction task where EnzRetro generates both SSREdits and their corresponding EC number directly from the product, reflecting the inherent relationship between enzymatic reaction transformation and enzyme specificity. Figure 4 shows the comparative results of the performance for EnzRetro, as compared to state‐of‐the‐art methods. EnzRetro (best) represents the highest‐performing instance of the model across multiple training runs, capturing the optimal configuration or epoch that yielded the best accuracy. In contrast, EnzRetro (mean) reflects the average performance of the model over five epochs of training, providing a robust measure of its typical or expected accuracy across repeated runs. We adopt the Top‐k exact match accuracy as the metric to measure the percentage of Top‐k predictions containing SSREdits and EC numbers that precisely match the ground truth in the ECREACT dataset. As shown in Figure 4, EnzRetro consistently outperforms Probst's method across all EC levels and accuracy metrics. For instance, at EC X.‐.‐.‐, EnzRetro (best) achieves 67% Top‐1 accuracy, while Probst's method is lower, about 61%. At EC X.X.X.X, EnzRetro (best) maintains 57.5% Top‐1 accuracy, while Probst's method drops to 53%. Furthermore, the performance gap widens for higher‐top metrics, with EnzRetro (best) achieving 89% Top‐10 accuracy at EC X.‐.‐.‐, compared to approximately 72% for Probst's method, surpassing it by 17%. These results highlight the effectiveness of the proposed method in capturing the relationship between products, reactants, and enzymatic functions, enabling more reliable predictions in complex scenarios. Besides, there is a drop in accuracy from EC X.‐.‐.‐ to EC X.X.X.X, pointing to a limitation in predicting highly specific enzymes due to the complexity of predicting SSREdits and EC number simultaneously. To conclude, by outperforming Probst's method, the proposed approach demonstrates its potential for real‐world applications, where accurate and simultaneous predictions of reactants and EC numbers are critical for understanding and designing enzymatic reactions.

#### 2.3.2 Analysis of Model Reasoning Process

**FIGURE 5:**
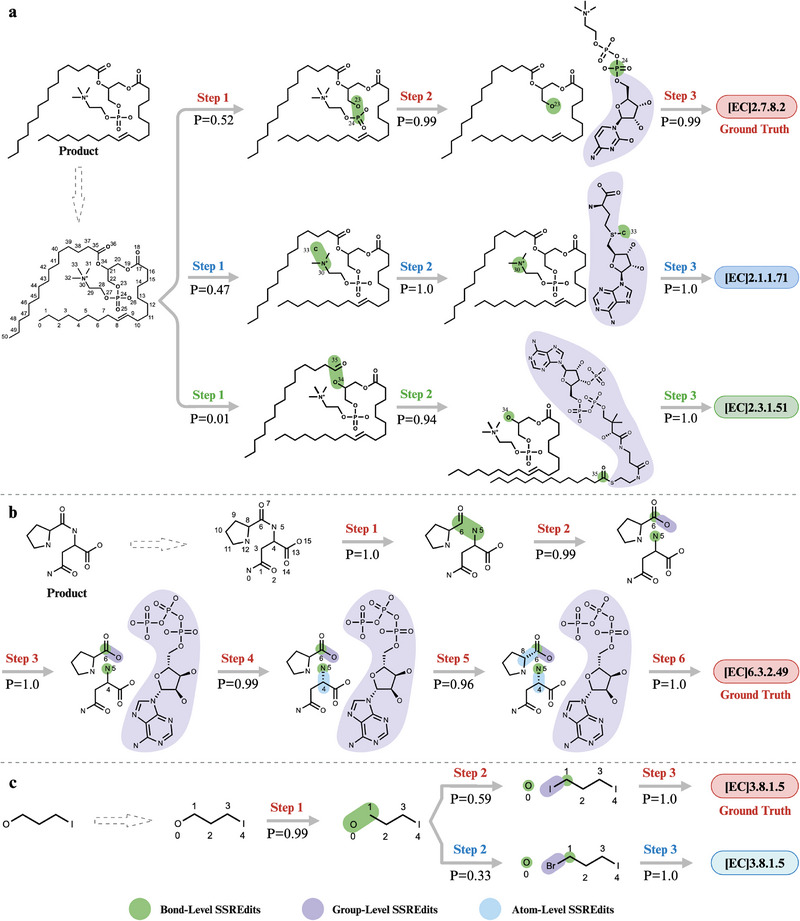
Retrosynthesis reasoning predictions by EnzRetro trained on the ECREACT dataset. First, the product is assigned corresponding IDs to each atom by the RDKit tool, and then encoded them to generate SSREdits for specific atoms, bonds, or subgroups. Next, the EC number of the enzyme is outputted sequentially by using the comprehensive information present within SSREdits based on the enzymatic SSREdits generation model. The probabilities P associated with each SSREdit step are the model's token‐wise confidence scores generated autoregressively.

To gain insights into the reasoning process of EnzRetro, we randomly select three reactions from the test set of ECREACT and visualize the generation process in Figure 5. First, the target products are assigned corresponding IDs to each atom by the RDKit tool, and then encoded them to generate SSREdits for specific atoms, bonds, or functional subgroups. Next, the EC number of the enzyme is outputted sequentially by using the comprehensive information present within SSREdits based on the enzymatic SSREdits generation model.

The first example in Figure 5a describes the hydrolysis of oxygen‐phosphorus bonds in phosphocholine groups. EnzRetro predicts the hydrolysis of an “OP” bond with a probability of 0.52 and the subsequent attachment of a phosphocholine group with a probability of 0.99. This reaction is catalyzed by enzymes classified under diacylglycerol cholinephosphotransferase (EC 2.7.8.2), corroborating the expected enzymatic classification. The Top‐2 prediction from EnzRetro generates feasible SSREdits corresponding to phosphatidyl‐N‐methylethanolamine N‐methyltransferase (EC 2.1.1.71), involving the cleavage of a “CN” bond with a probability of 0.47, along with the transfer of a methyl group and the incorporation of S‐adenosyl‐L‐homocysteine into the target product. It is worth noting that the Top‐2 results represent another set of feasible enzymatic SSREdits, reflecting the model's ability to generate multiple valid results.

The second example involves the enzymatic activity of L‐alanine–L‐anticapsin ligase (EC 6.3.2.49), as shown in Figure 5b, which catalyzes the cleavage of L‐alanine from the target product and the subsequent attachment of ATP. Despite the complexity of the generation process with six sequential steps, each step is executed with high precision, as evidenced by the consistently high probabilities associated with the predicted SSREdits.

Figure 5c illustrates the hydrolytic dehalogenation reactions catalyzed by haloalkane dehalogenase (EC 3.8.1.5). The process begins with the cleavage of the carbon–halogen (C–X) bond, which occurs with a high probability of 0.99. This step is followed by the attachment of a halide group, where the probability of incorporating an iodine group (“‐I”) is 0.59, and a bromine group (“‐Br”) is 0.33, highlighting the precision and versatility of haloalkane dehalogenase in mediating environmentally significant dehalogenation processes. These results underscore the robustness of EnzRetro in handling continuous inference edits, demonstrating its reliability in accurately predicting and executing multi‐step enzymatic transformations. More examples of predictions can be found in Figure S6, Supporting Information.

#### 2.3.3 Analysis of Incorrect Predictions

**FIGURE 6:**
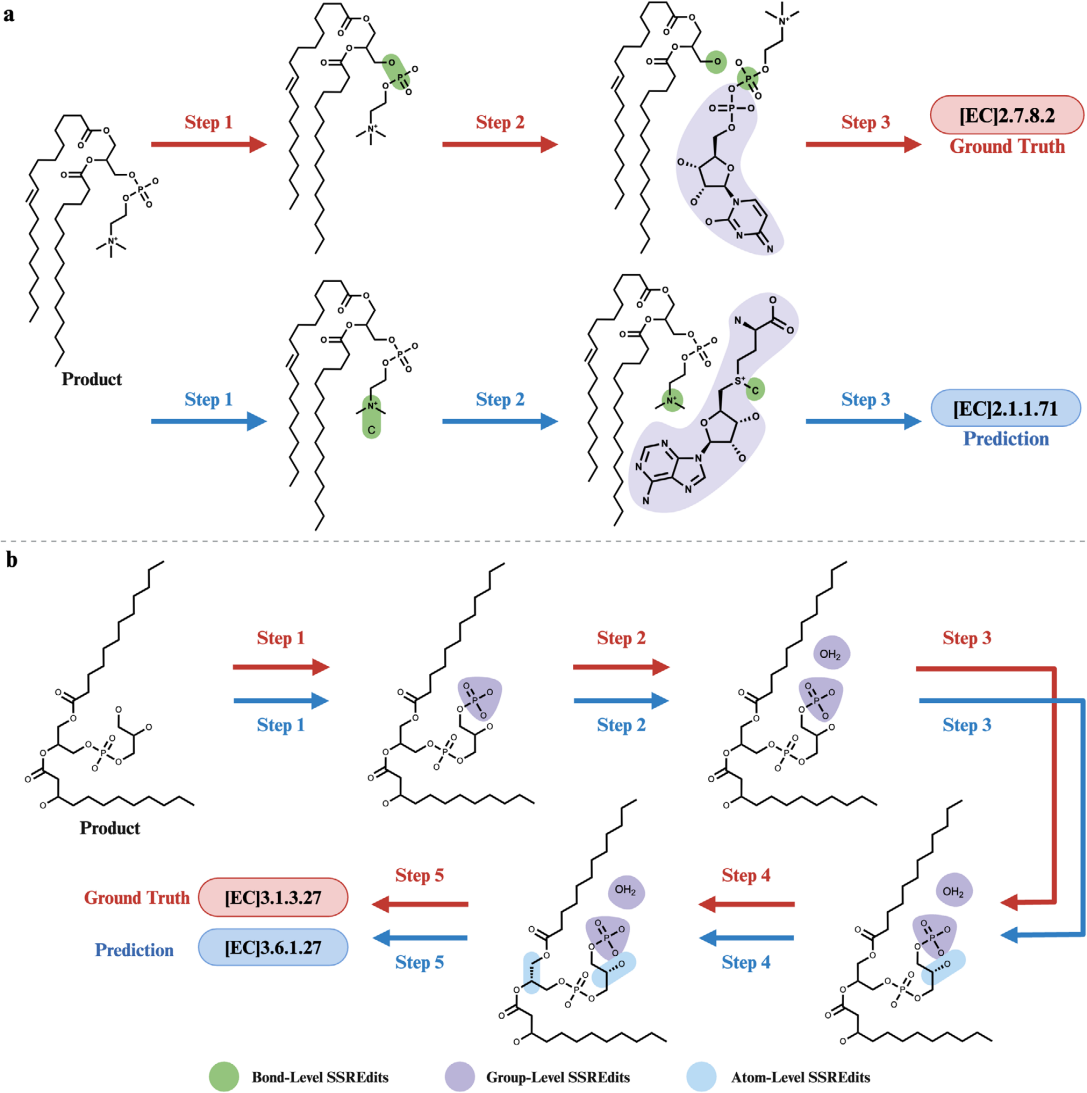
Examples of Top‐ranked prediction for different error categories by EnzRetro trained on the ECREACT dataset. (a) Multiple valid SSREdits and (b) EC number discrepancies for identical SSREdits.

To better understand the model's performance, we further investigate the incorrect predictions by EnzRetro on the ECREACT dataset. As shown in Figure 6, we observed that these errors can be categorized into two main types: (1) multiple valid SSREdits and (2) EC number discrepancies for identical SSREdits. In some cases, the predicted SSREdits differ from ground truth edits. However, the predicted SSREdits correspond to the alternative valid SSREdits also present in the dataset. This indicates that a single input can lead to multiple feasible solutions, reflecting the inherent flexibility in enzymatic transformations.

In the first example in Figure 6a, EnzRetro generates feasible SSREdits associated with phosphatidyl‐N‐methylethanolamine N‐methyltransferase (EC 2.1.1.71), which catalyzes the transfer of a methyl group and the incorporation of S‐adenosyl‐L‐homocysteine as a substructure into the target product. In contrast, the ground truth SSREdits correspond to diacylglycerol cholinephosphotransferase (EC 2.7.8.2), which catalyzes the hydrolysis of an “OP” bond and the transfer of a phosphocholine group to the substrate. This is not a failure of the proposed generative model but a reflection of the fundamental property of biochemical data itself. A single product molecule can often be transformed into different sets of valid reactants through distinct mechanistic pathways, each catalyzed by a different enzyme. The proposed model effectively captures this reaction diversity. Although it may exhibit reduced accuracy in single‐step retrosynthesis predictions, its generative nature enables the exploration of a more diverse set of candidate reactions and enhances the potential for discovering novel enzymatic pathways in multi‐step retrosynthesis planning.

In the second category, the predicted SSREdits are chemically accurate, yet the corresponding EC number differs from the ground truth. This error arises from an inherent challenge in the ECREACT dataset and the principles of enzyme classification. The EC number is assigned based on a combination of the reaction transformation and broader biochemical context, including substrate specificity, protein structure and cellular context. However, the proposed model operates under the reasonable but simplified assumption that reaction transformation patterns (SSREdits) are the primary determinant of EC numbers. This assumption holds in most cases but fails when identical transformations are catalyzed by evolutionarily distinct enzymes with different substrate preferences. For example, as shown in Figure 6b, phosphatidylglycerophosphatase (EC 3.1.3.27) and undecaprenyl‐diphosphate phosphatase (EC 3.6.1.27) catalyze similar hydrolysis reactions with the same SSREdits but are categorized under distinct EC numbers. Our model, which learns a strong mapping from reaction transformation to EC number, is consequently challenged by these cases where the same transformation is assigned multiple labels based on external biochemical factors. It provides valuable insights into significant room for improvement in our current method, particularly in terms of the broader biochemical context of reactions.

#### 2.3.4 Analysis of Model Accuracy Validation

To evaluate the enzymatic prediction accuracy of EnzRetro, we performed the retrosynthesis analysis on three biosynthesis pathways of significant industrial importance: the deracemization of primary amines [60], the production of n‐butanol [61] and the synthesis amorpha‐4,11‐diene [62]. These pathways are strictly excluded from our training dataset. By performing a sequential retrospective analysis on these established pathways, we aimed to verify whether the model could correctly identify the ground‐truth enzymatic transformations and EC numbers from the product inputs.

**FIGURE 7:**
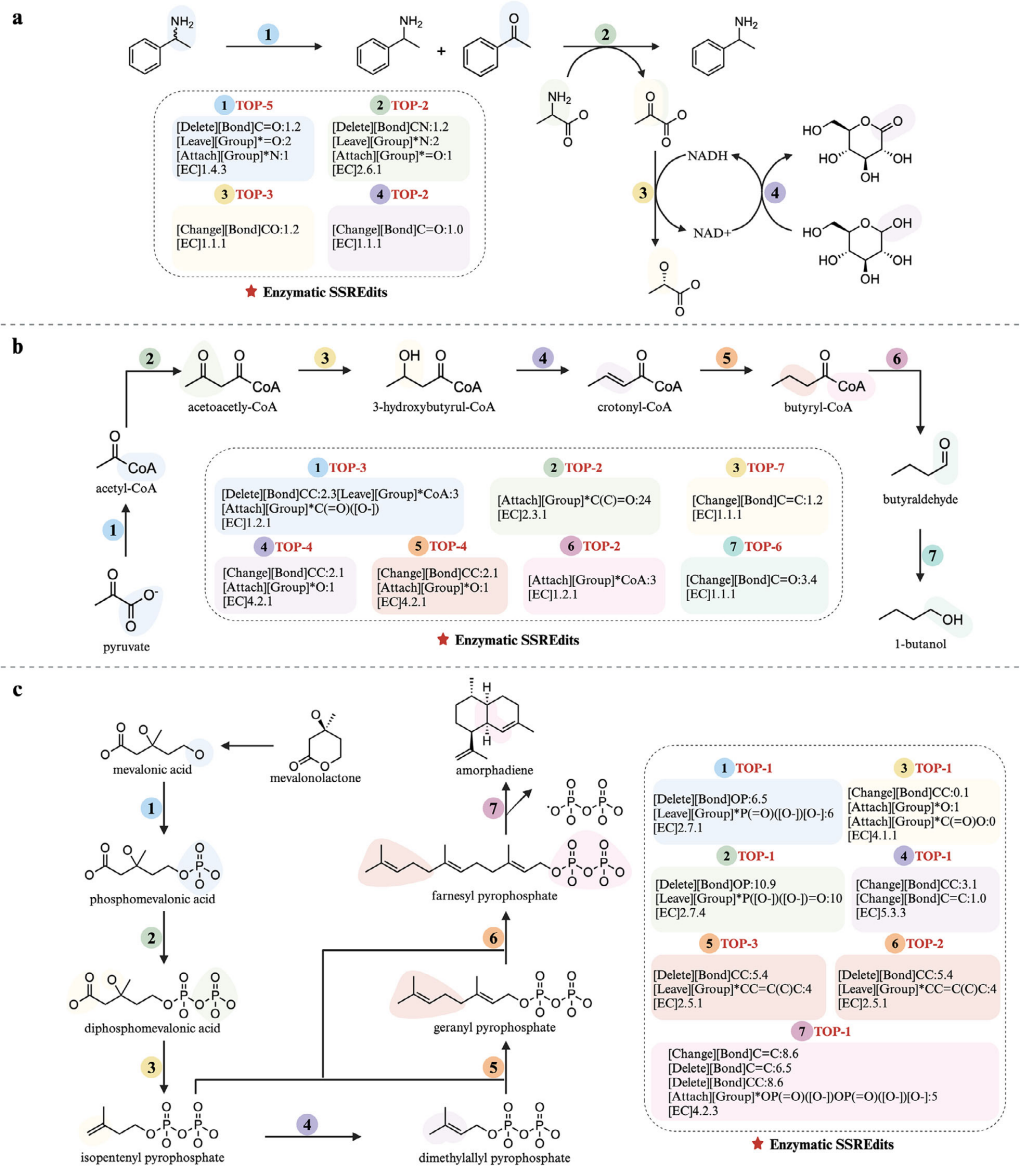
Enzymatic retrosynthesis planning by EnzRetro. (a) The deracemization of primary amines. (b) The production of n‐butanol. (c) The production of amorpha‐4,11‐diene. All enzymatic SSREdits are generated for retrosynthesis, while the pathways are presented in the forward direction, illustrating the sequential synthesis from the starting molecule to the final product.

The deracemization of primary amines is critical for synthesizing enantiomerically pure compounds, particularly for active pharmaceutical ingredients (APIs) where specific enantiomers exhibit distinct biological activities. Traditional chemical synthesis methods, such as kinetic resolution or dynamic kinetic resolution, often suffer from limitations like low yields or harsh conditions. To address these challenges, O'Reilly et al. [60] developed a cell‐free enzymatic pathway using a monoamine oxidase and transaminase, offering an efficient and sustainable alternative for deracemization. Reactions were performed with purified enzymes in 1 mL mixtures incubated at 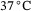 for 24–36 h. As shown in Figure 7a, EnzRetro accurately predicted this four‐step pathway with enzymes. In this enzymatic pathway, the process begins with selective oxidation, where a purified monoamine oxidase (EC 1.4.3.4) converts the methylbenzyl amine into the imine product by removing an amine group (‐NH2) from the substrate and forming an imine intermediate. Next, the transaminase (EC 2.6.1.‐) catalyzes the reductive amination of acetophenone, transferring an amino group from an appropriate donor to convert acetophenone into the desired amine. Then, pyruvate serves as the electron acceptor, while NADH donates electrons, converting pyruvate into L‐lactate and regenerating NAD+ through the enzyme lactate dehydrogenase (EC 1.1.1.‐). The glucose dehydrogenase (EC 1.1.1.‐) is the final enzyme in the cascade, aiming to recycle the costly NADH cofactor.

N‐butanol is a valuable biofuel and chemical feedstock with diverse industrial applications, and its biological production has gained significant interest as a sustainable alternative to petroleum‐based fuels [61]. While in vivo production faces challenges such as cellular toxicity and metabolic competition, cell‐free systems allow for rapid prototyping. The cell‐free system utilized *Escherichia coli* lysates, 200 mM glucose, and key cofactors in a 25 μL reaction at 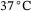 for 24 h. As illustrated in Figure 7b, our method successfully predicted the core seven‐step pathway from pyruvate to 1‐butanol. As illustrated in Figure 7b, our method successfully predicted the core components of the pathway, encompassing seven key reactions from pyruvate to 1‐butanol. In this enzymatic pathway, pyruvate is converted to produce acetyl‐CoA via pyruvate dehydrogenase (EC 1.2.1.‐), and then a heterologous thiolase enzyme (EC 2.3.1.‐) catalyzes the condensation of acetyl‐CoA to form acetoacetyl‐CoA. Next, the 3‐hydroxybutyryl‐CoA dehydrogenase (EC 1.1.1.‐) reduces acetoacetyl‐CoA to 3‐hydroxybutyryl‐CoA, using NADH as a cofactor, followed by the dehydration of 3‐hydroxybutyryl‐CoA into crotonyl‐CoA by crotonase (EC 4.2.1.‐). The fifth step involves the butyryl‐CoA dehydrogenase (EC 1.3.1.‐), converting crotonyl‐CoA into butyryl‐CoA through changing bond type. Then, the butanal dehydrogenase (EC 1.2.1.57) catalyzes the conversion of butyryl‐CoA to butyraldehyde, followed by reduction to n‐butanol with EC 1.1.1.‐. This modular approach, detailed in the cell‐free system, achieves n‐butanol titers up to 1.43 g/L, demonstrating high productivity and flexibility.

The production of artemisinin and its derivatives has been attracting considerable interest for the treatment of malaria, a major global health challenge [62]. Amorpha‐4,11‐diene, one of the important precursors to artemisinin, is a natural compound traditionally extracted from the plant Artemisia annua. However, the growing challenge of drug resistance calls for the development of non‐natural artemisinin derivatives to enhance the effectiveness of malaria treatment. Figure 7c shows a seven‐step pathway for the synthesis of amorpha‐4,11‐diene in a cell‐free system, reconstructing the mevalonate (MVA) pathway by a multienzyme system. Reactions with purified enzymes were carried out in 100 mm Tris/HCl (pH 7.4), 10 mm MgCl2, 10 mm mevalonic acid, and 15 mm ATP at 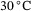. EnzRetro successfully predicted all seven reaction steps within the top three predictions. The first step uses a mevalonate kinase (EC 2.7.1.‐) to phosphorylate MVA to form phosphomevalonate (PMVA). Second, it converts PMVA into diphosphomevalonate (PPMVA) through phosphomevalonate kinase (EC 2.7.4.‐). Thirdly, PPMVA is decarboxylated by diphosphomevalonate decarboxylase (EC 4.1.1.‐) to produce isopentenyl pyrophosphate (IPP). Next, IPP is isomerized into dimethylallyl pyrophosphate (DMAPP) through isopentenyl pyrophosphate isomerase (EC 5.3.3.‐). In the following two steps, IPP and DMAPP are combined to synthesize farnesyl pyrophosphate (FPP) catalyzed by the same enzyme, farnesyl pyrophosphate synthase (EC 2.5.1.‐). Finally, amorpha‐4,11‐diene synthase (EC 4.2.3.‐) cyclizes FPP into amorpha‐4,11‐diene. The process begins with MVA as the starting substrate, which is sequentially transformed through the enzymatic steps to yield amorpha‐4,11‐diene. This in vitro system eliminates the need for living cells, allowing for direct manipulation of reaction conditions to optimize product yield, achieving nearly 100% efficiency for the desired amorpha‐4,11diene product.

To further evaluate the robustness of our framework, we benchmarked its performance against two established enzymatic retrosynthesis tools: BioNavi‐NP and RetroBioCat. The comparative results across the three case studies are illustrated in Figures S7–S9, Supporting Information. As shown in Figure S7, Supporting Information, baseline performance was sparse in this short cascade for the deracemization of primary amines. BioNavi‐NP could only identify the final reaction step (Top‐3), and RetroBioCat was limited to predicting the third step. In contrast, EnzRetro reconstructed the full sequence, notably predicting the final step with higher confidence (Top‐2) than BioNavi‐NP. For the n‐butanol production in Figure S8, Supporting Information, BioNavi‐NP demonstrated competitive accuracy on isolated reactions, achieving slightly higher rankings than EnzRetro on steps 2, 3, 5, and 7 (e.g., Top‐1 for steps 2 and 3). However, it failed to predict steps 1, 4, and 6, rendering the overall pathway incomplete. Similarly, RetroBioCat successfully identified the initial and final segments (steps 1–3 and 7) but failed to bridge the intermediate gap (steps 4–6). Conversely, EnzRetro successfully navigated the full seven‐step sequence without discontinuity. Besides, the comparison on the amorpha‐4,11‐diene pathway in Figure S9, Supporting Information, highlights the superior ranking precision of EnzRetro. While BioNavi‐NP successfully retrieved all seven steps of this pathway, its confidence levels were markedly lower, with only a single step predicted at Top‐1. However, EnzRetro demonstrated exceptional precision, identifying 5 out of 7 reactions as Top‐1 predictions. RetroBioCat failed to predict any steps within this complex pathway.

Overall, across the three enzymatic pathways, a total of 18 reaction steps, EnzRetro accurately predicted 13 steps within the Top‐3 rank (with five being Top‐1), while the remaining steps were predicted within the Top‐7 rank. These successful reconstructions highlight the model's strong generalization ability on unseen data. The results confirm that EnzRetro can not only accurately predict the structural transformations of enzymatic reactions but also correctly classify the associated EC numbers, validating its potential as a reliable tool for retrosynthetic planning.

### 2.4 Enzymatic Retrosynthesis Planning via Integrated Web Platform

To assess the practical utility of EnzRetro in multi‐step retrosynthesis planning, we developed an automated retrosynthesis tool accessible via a user‐friendly web platform. This platform integrates the multi‐objective optimization algorithm—adapted from our prior work [63]—to enable the design of complete enzymatic pathways through sequential retrosynthesis prediction. By modeling the search space as an AND‐OR tree and employing Monte Carlo tree search (MCTS), the system maintains a Pareto front of candidate pathways, simultaneously optimizing for multiple objectives. More details are described in Section 4.4.

**FIGURE 8:**
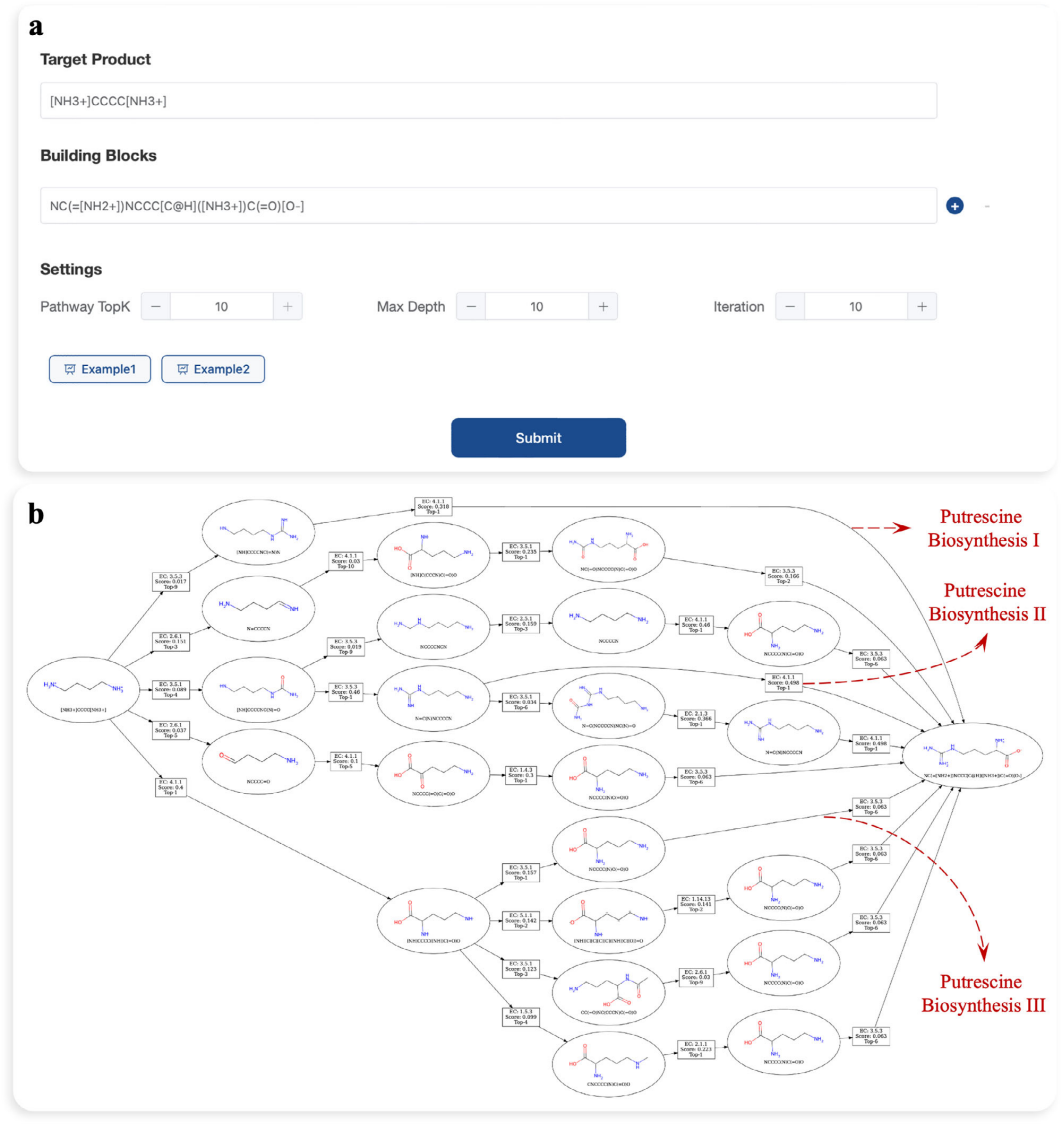
The interface and case study of EnzRetro web platform. (a) The input interface of EnzRetro web platform. (b) Predicted pathways of putrescine biosynthesis.

As shown in Figure 8a, the platform is designed for ease of use, requiring only the structure of the target molecule as a strict input. The diversity of output pathways is governed by the *Pathway Top‐K* parameter (default: 10), while the maximum pathway length is constrained by *Max Depth* (default: 10 steps). Advanced settings, including the list of available building blocks, can be modified to align with specific experimental constraints.

The predicted biosynthetic pathways are rendered as an interactive AND‐OR graph, where OR‐nodes represent molecules (circular nodes) and AND‐nodes denote enzymatic reactions with EC numbers (square nodes). Users can trace the target molecule back to potential starting materials through various branching pathways. Clicking on any node reveals a detailed information panel on the right, displaying specific molecular structures, enzyme EC numbers, and the predicted cost associated with each step.

To validate EnzRetro's capability, we conducted a case study on the biosynthesis of putrescine from L‐arginine, which is essential for normal cell growth and well‐known for its multiple parallel biosynthetic pathways. As illustrated in Figure 8b, EnzRetro successfully navigated the complex search space to reconstruct a comprehensive network containing three distinct canonical pathways. For putrescine biosynthesis I [64], EnzRetro correctly identified the initial decarboxylation of L‐arginine to agmatine (EC 4.1.1.19), followed by the hydrolytic conversion to putrescine (EC 3.5.3.11). In parallel, putrescine biosynthesis II [65] accurately reconstructed the three‐step sequence by EnzRetro, involving the conversion of L‐arginine to agmatine (EC 4.1.1.19), subsequent processing to N‐carbamoylputrescine (EC 3.5.3.12), and final hydrolysis to putrescine (EC 3.5.1.53). Additionally, EnzRetro successfully predicted putrescine biosynthesis III [66], including hydrolysis of L‐arginine to L‐ornithine (EC 3.5.3.1), followed by decarboxylation to yield putrescine (EC 4.1.1.17). In contrast, when evaluated under identical settings to assess performance with state‐of‐the‐art tools, BioNavi‐NP identified only a single pathway (putrescine biosynthesis III), while RetroBioCat failed to generate any valid predictions.

These results demonstrate the superiority of EnzRetro in multi‐step retrosynthesis planning and pathway diversity, effectively reconstructing biological ground truths without manual intervention.

## 3 Discussion

EnzRetro represents a foundational tool for synthetic biology and metabolic engineering, bridging the gap between computational pathway design and practical enzymatic implementation. By integrating pathway synthesis and enzyme identification into a unified, interpretable, and efficient framework, EnzRetro paves the way for scalable and rational design of enzymatic pathways. By introducing SSREdits, a novel approach to dynamically represent structural transformations at specific enzyme active sites, EnzRetro enhances the efficiency, accuracy, and interpretability of enzymatic retrosynthesis planning, offering a powerful tool and enabling a more cohesive and efficient design process. We apply pretrain‐finetune architecture and employ three pretraining tasks that facilitate learning meaningful representations of SSREdits. After that, two specific models are created through fine‐tuning the pretrained model. Specifically, the SSREdits generation model is developed to predict the possible sequence of edits at specific sites and sequentially generate the reactants. In contrast to previous methods, which require additional generative modules (as in MEGAN) or predefined substructure selection (as in Graph2Edits), EnzRetro distinguishes itself by its capacity to directly predict the reaction edits at specific sites and autonomously generate the substructures that need to be added or deleted. EnzRetro achieves competitive performance on USPTO‐50k and USPTO‐MIT datasets for retrosynthesis and forward synthesis, respectively. Additionally, the enzymatic SSREdits generation model is introduced to predict the corresponding EC number for enzymatic reactions by integrating rich and targeted information from reaction edits at specific sites. Comprehensive evaluations on the benchmark dataset ECREACT demonstrate that our method achieves a promising 97.7% Top‐1 exact accuracies and shows comparable performance compared to the other state‐of‐the‐art models. Finally, the one‐pot learning approach of EnzRetro further distinguishes it from prior frameworks. EnzRetro generates enzymatic SSREdits in an auto‐regressive way, to combine pathway synthesis and enzyme identification into an ont‐pot leaning and reduce computational overhead and enhance interpretability. Extensive experiments on three enzymatic pathways confirmed EnzRetro's precision in simultaneously predicting structural transformations and EC numbers. We extended this capability to multi‐step retrosynthesis planning based on a multi‐objective optimization framework deployed on an interactive web platform. This integrated system effectively mitigates error propagation and ensures pathway diversity, as evidenced by the successful reconstruction of multiple putrescine biosynthetic pathways that outperformed existing baselines. These advances establish EnzRetro as a robust platform for data‐driven metabolic engineering and a catalyst for progress in green chemistry and sustainable biomanufacturing.

Although EnzRetro shows promising performance for enzymatic retrosynthesis, there remain several challenges that need to be addressed to facilitate its widespread application. A primary constraint is data scarcity, particularly for highly specific enzyme classes at the EC X.X.X.X level, where accuracy is limited by the long‐tailed distribution of enzymatic reaction data. To overcome this, we plan to employ advanced transfer learning techniques, hierarchical multi‐task learning across EC levels, and incorporation of protein sequence representations to transfer functional knowledge to data‐scarce enzymes. Furthermore, the current model focuses exclusively on reaction transformation and does not yet account for kinetic parameters, reaction conditions, or enzyme stability, which are critical for practical pathway implementation. Extending EnzRetro to predict or rank pathways by thermodynamic and kinetic feasibility represents a key next step. Finally, while these cases demonstrate conceptual feasibility, the ultimate validation of any in silico pathway design is experimental. A key future goal is to establish a closed‐loop, iterative workflow where EnzRetro's predictions are rigorously tested, and the experimental results are fed back to further refine and validate the model, bridging the gap between computational prediction and practical biocatalysis.

## 4 Method

### 4.1 Details of Enzymatic SSREdits

There are two key components in the proposed SSREdits: (1) the action types for reaction transformation, and (2) site‐specific SMILES representations of molecular substructures. The action types include eight defined operations, as detailed in Table S2, Supporting Information: (1) delete bond, (2) change bond, (3) add bond, (4) attach group, (5) leave group, (6) change atom, (7) terminate, and (8) EC. These first seven actions collectively describe the stepwise transformations occurring during an enzymatic reaction. By encoding actions as tokens, the model can effectively learn and replicate the sequential nature of reaction transformations, enabling precise and structured prediction of chemical changes. The site‐specific SMILES representations of molecular substructures vary dynamically based on the input provided to the model. Importantly, these site‐specific SMILES are not derived from the training dataset nor predefined by domain experts. Instead, they are generated adaptively by the model, reflecting its capacity to generalize and infer reaction‐specific features without reliance on manual annotations or pre‐existing rules. This approach enhances the flexibility and predictive power of the framework. Instead of framing EC number prediction tasks as a multilabel classification problem in previously developed machine learning algorithms, EnzRetro predicts the EC number by employing an autoregressive model to learn the embedding space of product and reaction edits. Specifically, an additional action [EC] is introduced to predict the EC number corresponding to the reaction based on the predicted SSREdits. This action enables the model to associate specific reaction transformations with their catalyzing enzymes, facilitating accurate EC number prediction as part of the generative process. According to the generated enzymatic SSREdits, the product molecule can be sequentially converted into reactants, guided by the catalytic enzyme.

The examples of enzymatic SSREdits are shown in Figure. Before processing, the input products are assigned atom‐specific numbering using RDKit. This numbering ensures consistency and facilitates accurate identification of atomic sites involved in the reaction transformations during subsequent steps. The first edit [Delete][Bond]CN: 7.8 represents breaking down bond CN between atoms 7 and 8. And then, the edit is [Attach][Group]*[S@@+](CC[C@H](N)C(=O)O)C[C@H]1O[C@@H](n2cnc3c(N)ncnc32)[C@H](O)[C@@H]1O: 8, which refers to attaching functional substructure to atom 8. Next, the atom edit [Change][Atom]N: 7 is operated on atom 7 to change the charge of the product. To ensure the validity of the predicted molecule, we employ the [Terminate] edit action to adjust the hydrogen atoms in the product. This step ensures that the final molecular structure adheres to chemical valency rules and represents a chemically valid product. Finally, we apply the edit [EC] to output the EC number sequentially, using the comprehensive information present within SSREdits to accurately predict the corresponding enzyme.

### 4.2 Pretraining Tasks

To enhance the model's ability to learn the meaningful representation of SMILES and SSREdits, three pretraining tasks have been proposed as shown in Supplementary Figure 1. The first task enables the model to learn the fundamental rules and implicit patterns of SMILES. The latter two tasks help the model learn the underlying features of molecular transformations and the relationship between the SMILES modifications and reaction edits at specific sites. We use unlabeled molecular data from the PUBChem database [67] as the training dataset, which is represented using the SMILES format, and then tokenized into individual tokens for input into the model.

1. **FillMASK**: In this task, we randomly replace certain tokens in the given SMILES sequence. Specifically, we replace a segment of one to four tokens with the special token “[MASK]” while freezing the tokens preceding the “[MASK]” token, meaning they remain unchanged to preserve contextual information. This replacement operation is repeated iteratively, either until no token is left after the last “[MASK]” or until a maximum of five iterations is reached. The goal of this task is to predict the masked tokens effectively, improving the model's understanding of the SMILES sequences for the molecules.
2. **MakeSSREdits**: In this task, the original SMILES sequence of the target molecule is transformed into an edited SMILES sequence using randomly selected SSREdits from a predefined set. Then, the sequence of the original SMILES and edited SMILES that are connected by two greater‐than symbols is provided as input into the model to predict the applied SSREdits. The aim of this task is to train the model to understand and identify structural transformations in SMILES representations, thereby enhancing its capability to learn transformations and relationships between molecular structures.
3. **MakeSMILES**: The final task processes the original SMILES sequence in the same way as the “MakeSSREdits” task. This task uses the sequence of the original SMILES and SSREdits as input into the model, aiming to reconstruct modified molecular structures based on specific edits, thereby enhancing its ability to model molecular transformations and synthesize structural variations.

By pretraining the model on a large molecular database using these tasks, it effectively captures comprehensive information about molecular structure and reaction transformation. These tasks provide a robust foundation for fine‐tuning the model on downstream tasks, enabling it to generalize well across various applications.

### 4.3 Model Architecture and Training Details

The EnzRetro model is built upon a standard encoder–decoder architecture that incorporates relative position encoding as implemented in the text‐to‐text transfer transformer (T5)[68]. While GNNs are well‐suited for capturing molecular topology, our task demands not only structural comprehension but also the generative sequencing of structured sequential representations (SMILES and reaction edits). Sequence‐based models are particularly powerful to leverage large‐scale pretraining on molecular databases, and facilitate effective transfer learning to specialized enzymatic reaction databases like ECREACT. In contrast to pure decoder models (e.g., GPT), which rely on unidirectional self‐attention and simple contextual concatenation, the encoder–decoder architecture offers distinct advantages for structured generative tasks. It enables thorough comprehension of complex inputs and sequential generation of output. A key strength lies in its cross‐attention mechanism, which allows each decoding step to dynamically attend to all encoded input tokens, thereby enhancing the model's ability to capture input–output relationships. The T5 model has further demonstrated state‐of‐the‐art performance on a variety of Seq2Seq tasks in chemistry, including reaction prediction and retrosynthesis, underscoring its suitability for our proposed framework.

The T5 architecture consists of a six‐block encoder and a six‐block decoder, which can efficiently handle contexts of up to 256 tokens. We set the model dimension size to 256 to balance computational efficiency and model performance. Each block consists of a multi‐head self‐attention layer and a feed‐forward network (FFN). The multi‐head attention layer is constructed with 12 attention heads, each with a size of 64. The dimension of the FFN is set to 2048. To optimize the impact of relative positional information on model performance, we configure the number of relative attention buckets to 32 and the maximum relative attention distance to 128. Models are trained by using the AdamW optimizer with $\beta_{1}=0.9$ and $\beta_{2}=0.999$ for gradient decent optimization, along with a linear warmup to a peak learning rate of 5 $\times \;10^{-5}$. During model training, the dropout rate is set to 10% and the learning rate decays 2% after every 10,000 training steps.

All model training and inference were conducted on a server with two NVIDIA GeForce RTX 3090 GPUs. The pretraining process on the PUBChem database [67] required approximately 150 h. For downstream tasks, we trained the model until convergence and selected the checkpoint with the best validation performance to evaluate the final metrics. Fine‐tuning for retro prediction on the USPTO‐50k dataset took about 10 h, while forward prediction on the USPTO‐MIT dataset required approximately 20 h. Owing to the smaller scale of the ECREACT dataset, fine‐tuning for EC number prediction typically converged within a few hours ( 3–5 h). For retro prediction on the ECREACT datasets, due to the limited size of the dataset, the validation set was also included in the training process. Ultimately, 90% of the data was used for training, while 10% was reserved for testing. The model was trained for 10 epochs, and the average performance metrics from the last five checkpoints were calculated as the final reference result. For the comparative experiment between Product + SSREdits and Product + Reactants, the model was trained for five epochs, and the checkpoint with the best validation performance was selected for testing and final metric evaluation.

During inference on a single RTX 3090 GPU, our model demonstrates high throughput. Using beam search (beam size = 10), it processes approximately 1120 tokens per second. Given that EC number sequences are typically very short (<15 tokens) and most reaction‐related sequences are under 512 tokens, the inference for a single prediction is highly efficient. The moderate resource requirements and fast inference speed underscore the potential of EnzRetro for scalable deployment in bio‐catalytic pathway design.

### 4.4 Multi‐Step Retrosynthesis Planning as a Multi‐Objective Optimization Problem

Building upon our prior work [63], which formulated biosynthetic pathway design as a multi‐objective optimization problem, we adapted and extended this framework specifically for enzymatic retrosynthesis planning. The framework consists of four core components, designed to efficiently navigate the complex synthetic space and identify feasible pathways:

1. Representation: Given EnzRetro's proficiency in single‐step predictions, we model the synthetic pathway as an AND–OR tree, which outputs both precursor molecules and their associated EC numbers. An AND node represents a complete enzymatic reaction, including the set of precursor molecules (reactants) and the specific EC number. An OR node represents a molecule, capturing the multiple valid sets of reactants. The root node of the tree is the target molecule, and the leaf nodes are usually commercially available starting materials.
2. Selection: The search progression is guided by the multi‐objective optimization. We employ an MCTS algorithm to efficiently navigate the AND–OR tree. Rather than converging on a single scalar metric, the algorithm maintains and explores a Pareto front of candidate pathways, ensuring a diverse set of solutions that represent the best possible trade‐offs between competing objectives.
3. Expansion: For a given compound (OR‐node), EnzRetro performs single‐step retrosynthesis to yield sets of precursors from top‐k enzymatic SSREdits predictions using the beam search procedure. Each prediction, comprising both reactants and an EC number, is used to create a new AND‐node (the reaction with EC number) and subsequent OR‐nodes (the new precursor targets).
4. Evaluation: Candidate pathways are evaluated using a set of objective functions that assess their synthetic feasibility from multiple dimensions. A comprehensive evaluation typically considers both global pathway properties and local step‐wise attributes.

In this work, EnzRetro was trained on the ECREACT dataset at EC X.X.X.‐ for single‐step retrosynthesis. We evaluate enzymatic retrosynthesis pathways using three specific metrics. (1) Pathway length, representing the total reaction steps from the target molecule to starting materials. (2) Retrosynthetic accessibility score (RAscore), reflecting the EnzRetro model's probabilistic confidence for each single‐step retrosynthetic prediction. (3) Substrate similarity, calculating the Tanimoto similarity between the generated precursors and the best‐matching entry in our predefined starting material database. For the latter two step‐wise metrics, we compute a weighted average across all steps in a pathway to obtain aggregate scores for multi‐objective comparison. Then, we consider the multi‐step retrosynthesis planning as a multi‐objective optimization problem that includes three optimization objectives: minimizing the overall pathway length, maximizing the average RAscore, and maximizing the substrate similarity. Given the target molecule and its initial set of commercially available precursors, multi‐step retrosynthetic evaluation is performed using a beam size of 10, maximum pathway length of 10, and 100 MCTS iterations. The process terminates when a leaf node corresponds to one of the starting materials, indicating a complete pathway has been found, or upon reaching maximum number of iterations.

## Author Contributions

**Yahui Cao**: conceptualization, methodology design, writing – original draft. **Haoshu Chen**: dataset preparation, model training, and validation.

## Funding

This work was supported by the National Key Research and Development Program of China (Grant No.2025YFA0922300).

## Conflicts of Interest

The authors declare no conflicts of interest.

## Supporting information


**Supporting File 1**: exp270129‐sup‐0001‐SuppMat.docx.

## Data Availability

All the data used in this paper are publicly available and can be accessed from various sources. The USPTO‐50K dataset for retrosynthesis prediction is publicly available at https://github.com/Jamson‐Zhong/Graph2Edits. The USPTO‐MIT dataset for forward prediction is available at https://github.com/HelloJocelynLu/t5chem.git. The ECREACT dataset is publicly available at https://github.com/rxn4chemistry/biocatalysis‐model. The BioCyc database is publicly available at https://biocyc.org/. EnzRetro can be accessed freely as the web server http://bdatju.com. The source code of this work and associated trained models are available at GitHub: https://github.com/bellacaoyh/enzretro. Detailed Python notebooks for replicating all calculations and accessing trained model checkpoints are provided on the corresponding GitHub page.
