# Supplementary material for: EnzRetro: Enzymatic Retrosynthetic Planning With Site‐Specific Reaction Edits Based on Sequence Generative Architecture
Source: Exploration (Beijing). 2026 Feb 26;6(2):70129. doi: 10.1002/exp2.70129 (PMC13094528; doi:10.1002/exp2.70129)
Supplement: Supplementary file 1 — Supporting File 1: exp270129‐sup‐0001‐SuppMat.docx. [file EXP2-6-70129-s001.docx]

**Supplementary Information:**

**EnzRetro: Enzymatic Retrosynthetic Planning with Site-specific Reaction Edits based on Sequence Generative Architecture**

**Supplementary Figures**


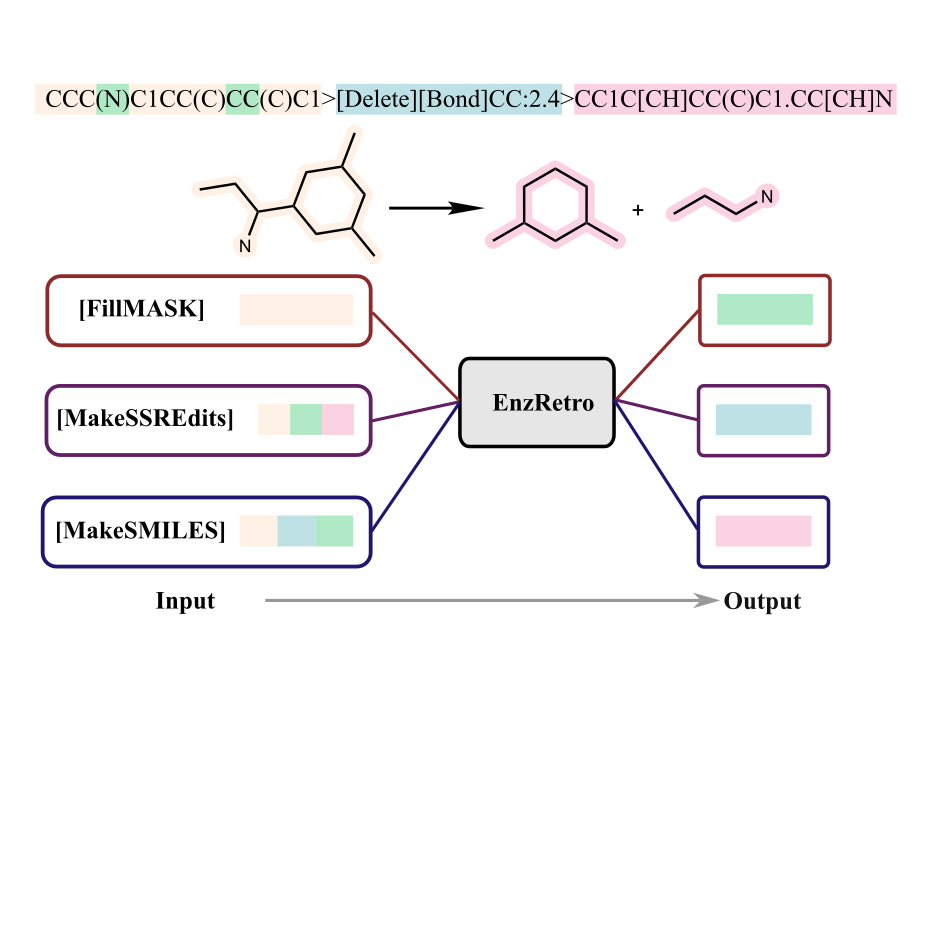


**Supplementary Figure 1. Overview of pretraining tasks for EnzRetro based on PubChem database.** The first task enables the model to learn the representation of SMILES. The latter two tasks help the model learn the features of molecular transformations and the relationship between the SMILES modifications and SSREdits.


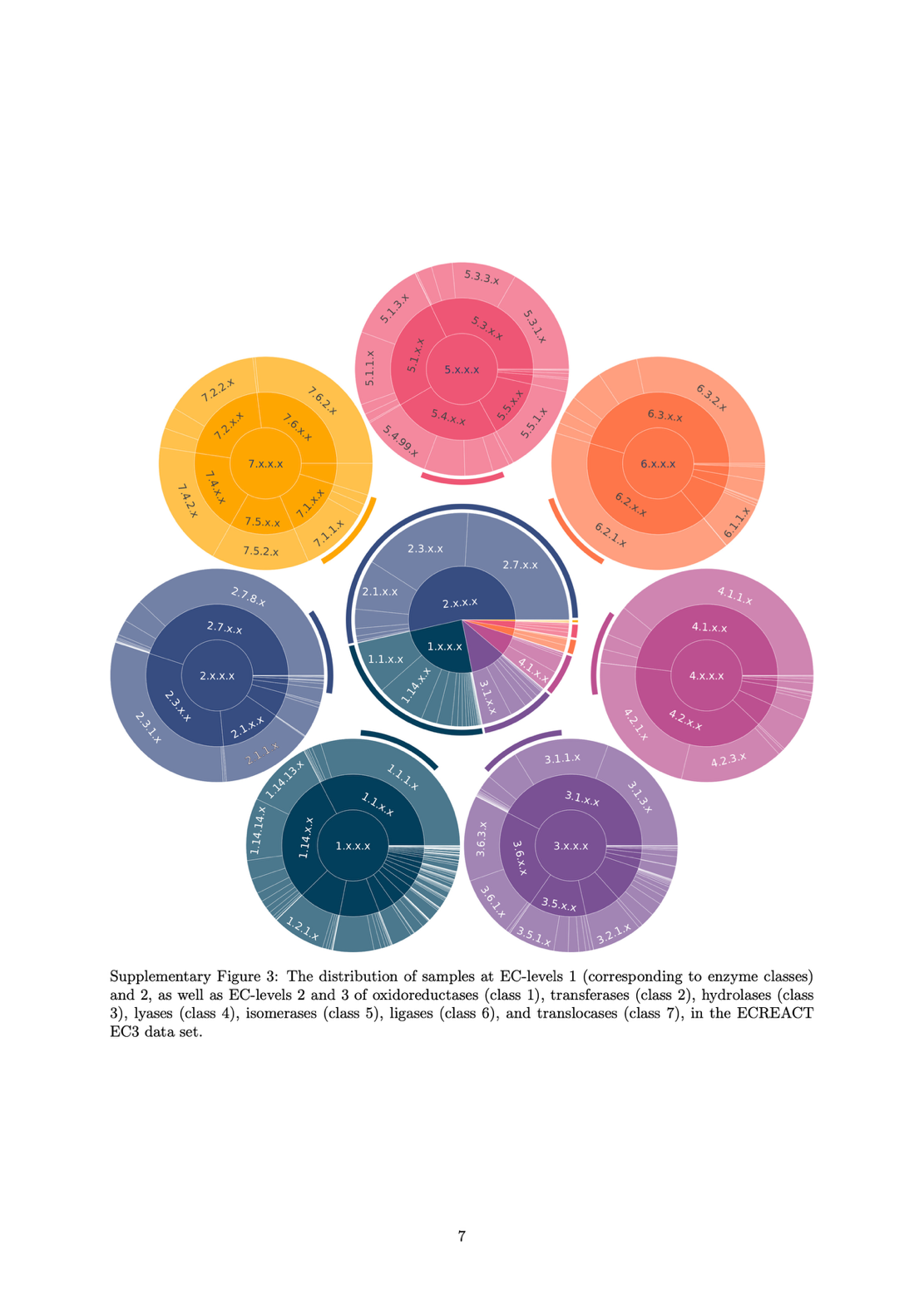


**Supplementary Figure 2. The distribution of samples across different EC levels in the ECREACT**^[1]^**.**


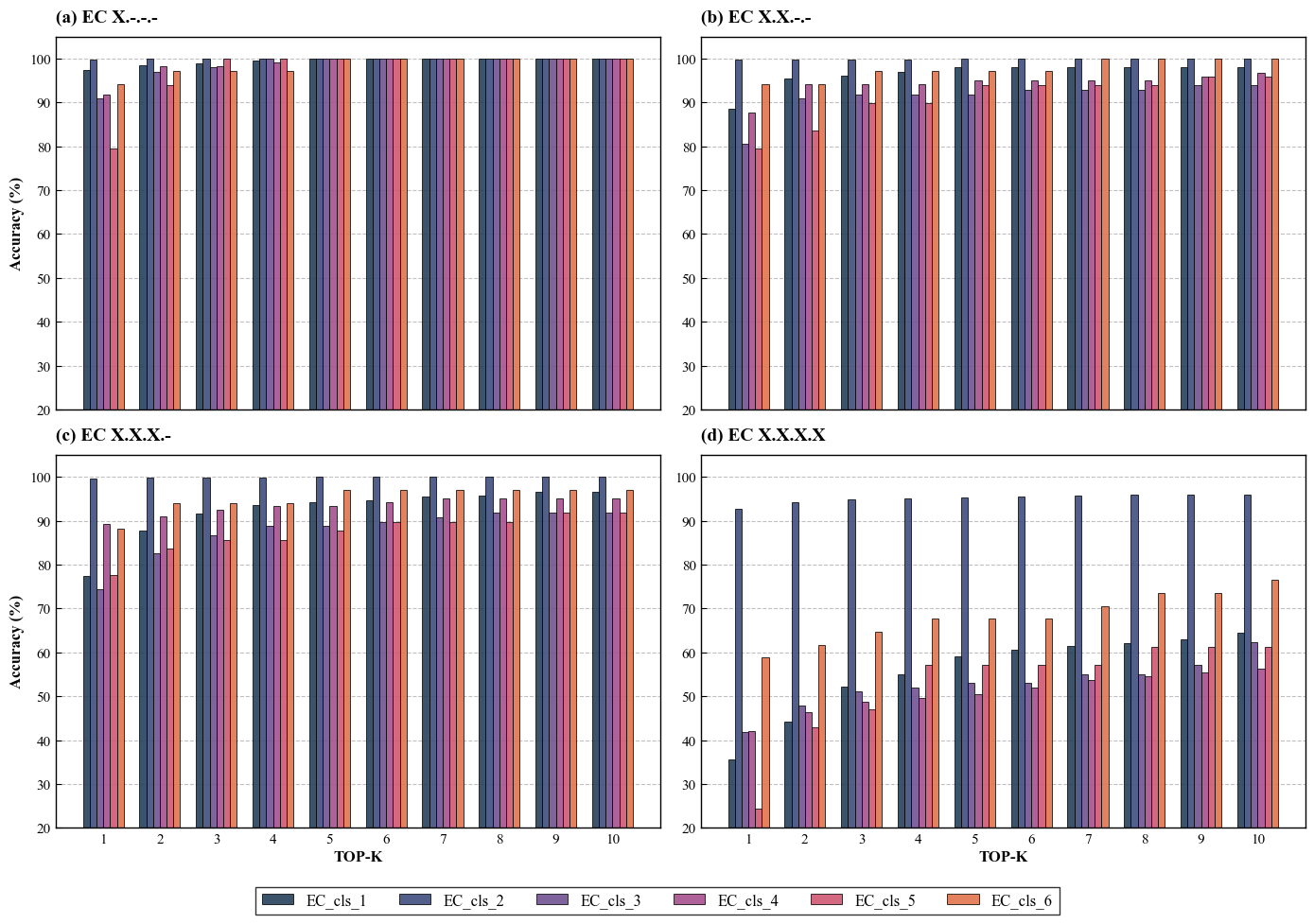


**Supplementary Figure 3. Class-wise performance evaluation across different levels of EC number (from EC X.-.-.- to EC X.X.X.X).** The subplots display the TOP-K accuracy trends for six individual classes (EC_cls_1 to EC_cls_6) within: (a) EC X.-.-.-, (b) EC X.X.-.-, (c) EC X.X.X.-, and (d) EC X.X.X.X. Each bar group represents the accuracy metrics from TOP-1 to TOP-10.


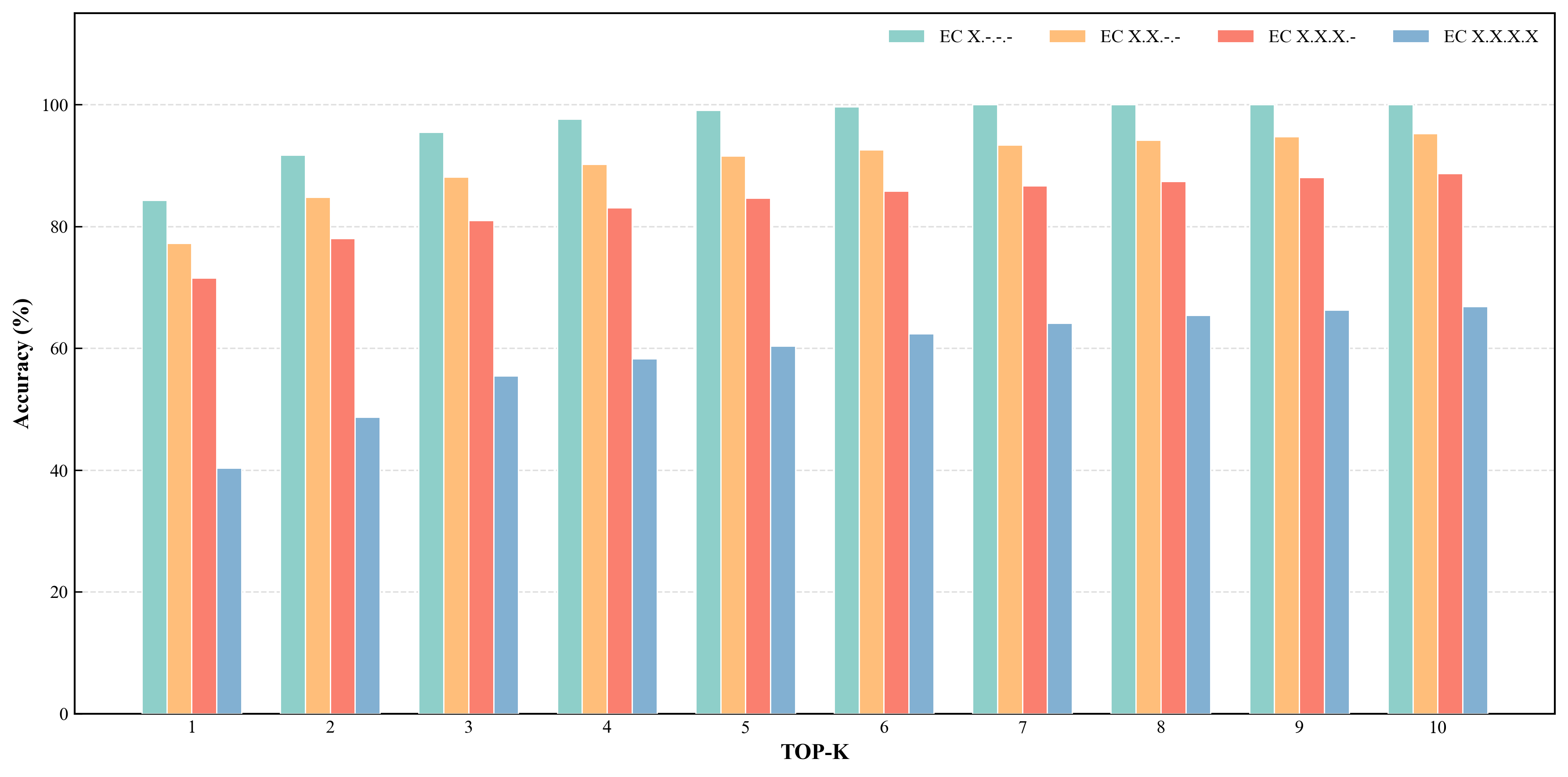


**Supplementary Figure 4. Comparison of TOP-K prediction accuracy on BioCyc database**^[2]^ **across different levels of EC number (from X.-.-.- to EC X.X.X.X).** The bar chart illustrates the cumulative accuracy from TOP-1 to TOP-10. The $x$-axis represents the k value, while the $y$-axis denotes the percentage of accuracy. Different colors and hatching patterns distinguish the four levels of EC number.


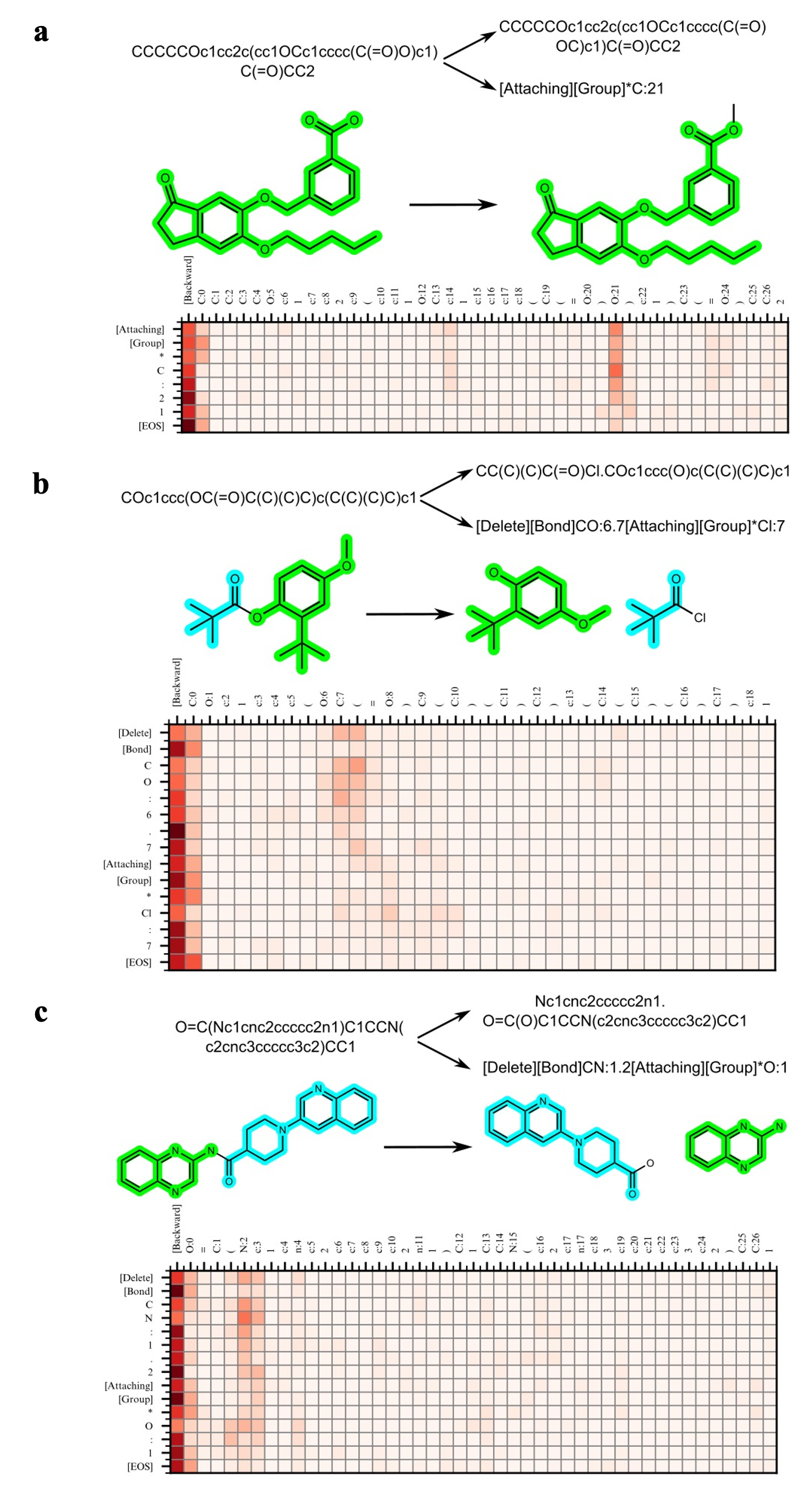


**Supplementary Figure 5****. Attention weights interpretation for the SSREdits generation model trained on USPTO-50k dataset.** The horizontal axis represents the tokens of target molecule, and the vertical axis refers to the output of the SSREdits. The darker the token, the more attention a specific token has received in that particular layer or output step. The colouring on the chemical reaction visually maps the transitions from reactants to the product in the ground truth.

**a**

**b**

**c**


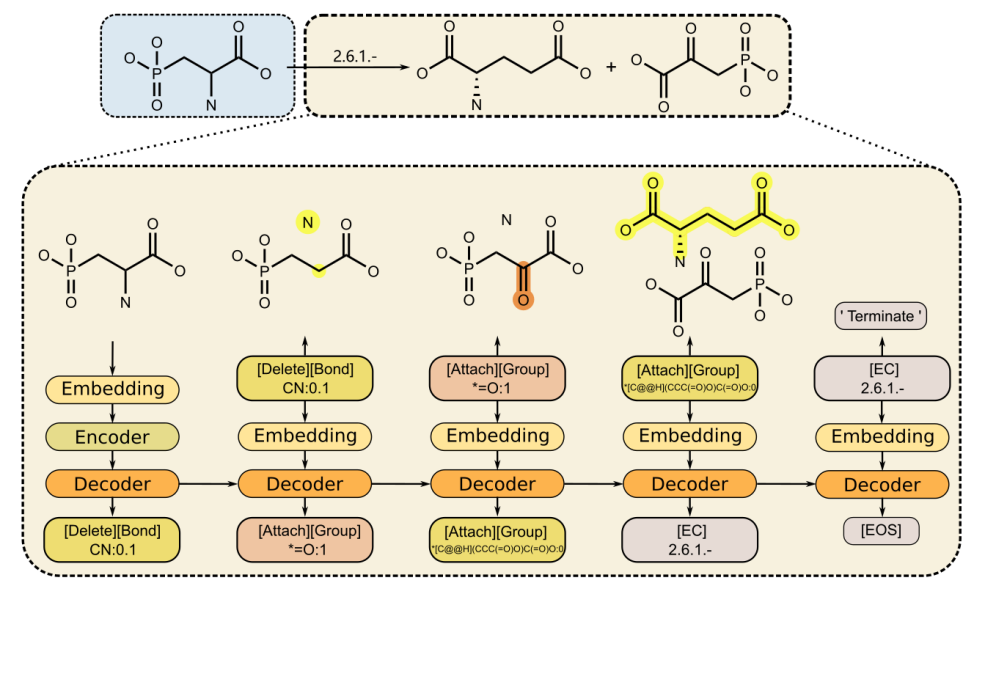

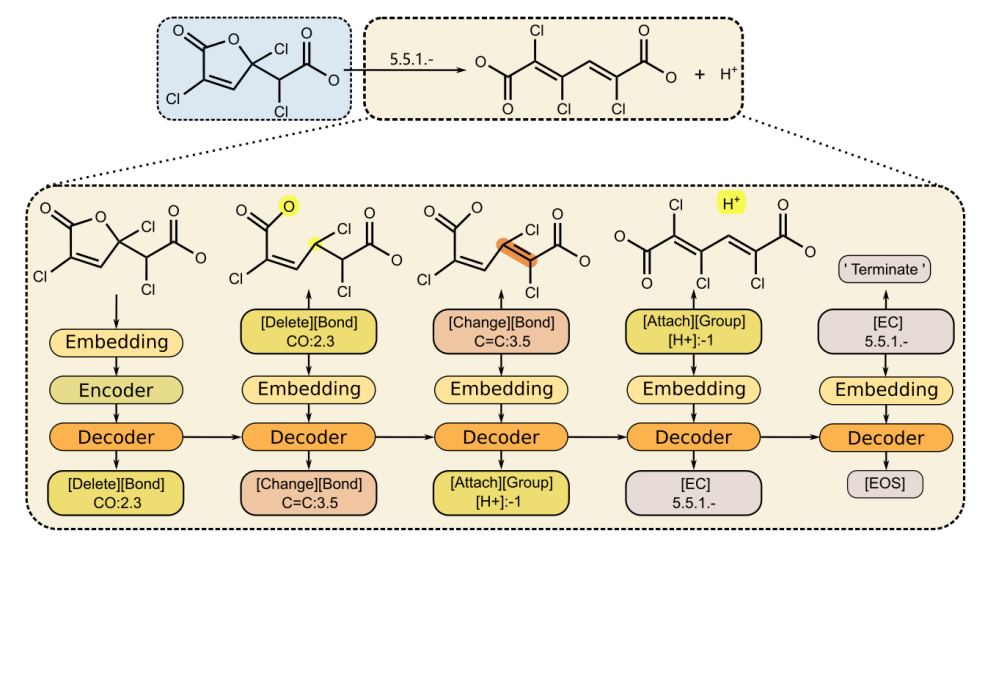

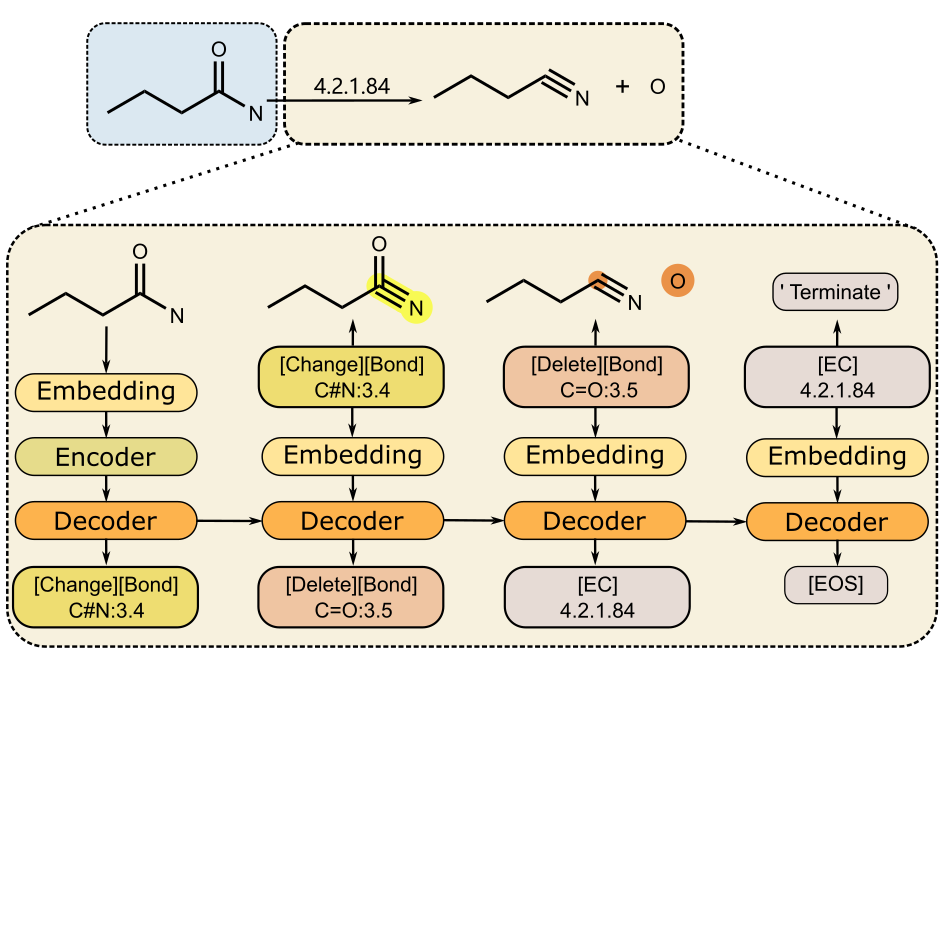


**Supplementary Figure 6. More examples of single-step retrosynthesis reason prediction by EnzRetro trained on the ECREACT dataset.**


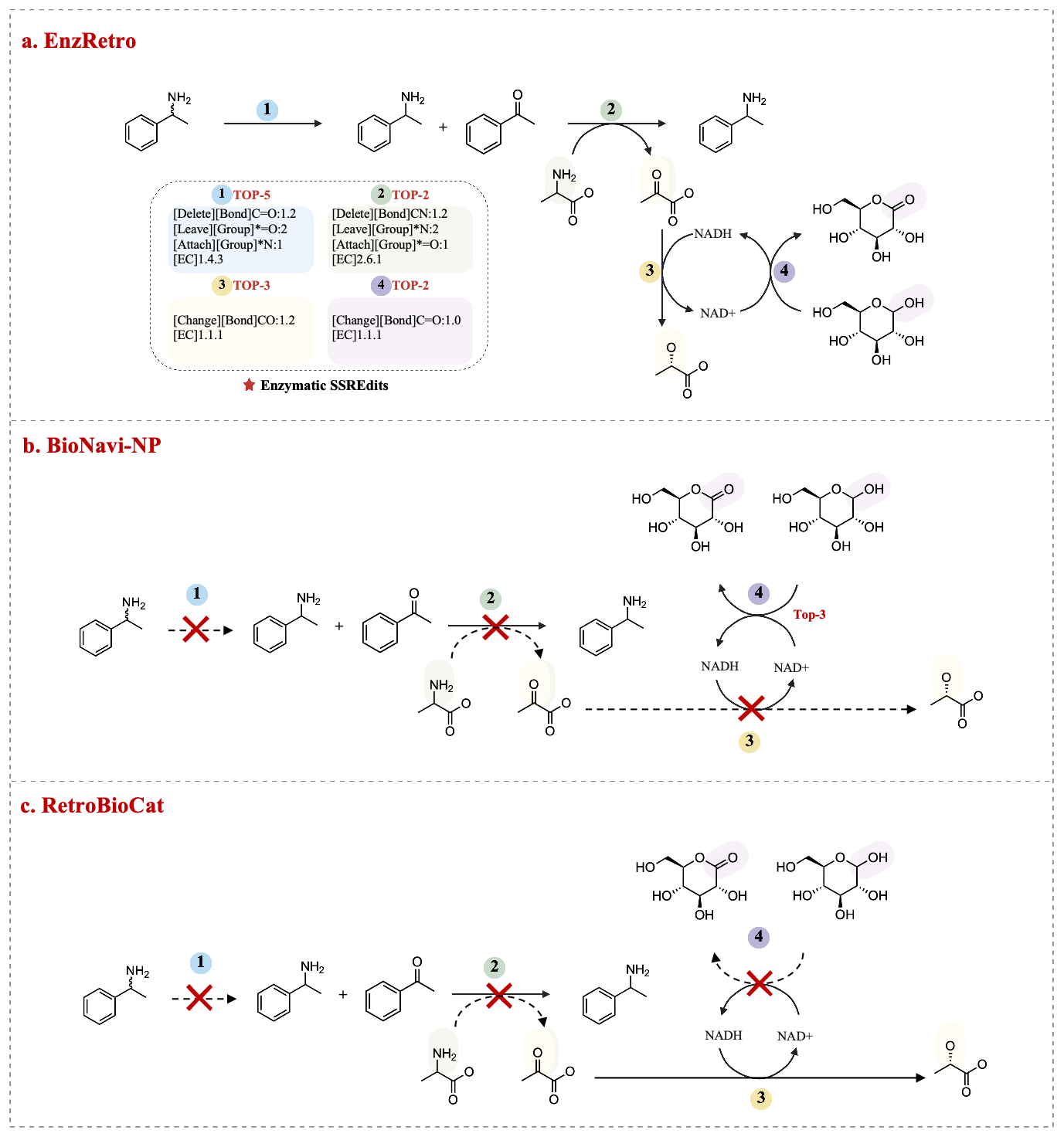


**Supplementary Figure 7. Comparative analysis of enzymatic retrosynthesis planning for the deracemization of primary amines.** The pathways are generated by (a) EnzRetro, (b) BioNavi-NP^[3]^ and (c) RetroBioCat^[4]^, respectively. EnzRetro successfully identifies a complete and feasible pathway. In contrast, dashed lines marked with a red cross denote reaction steps that the corresponding tools failed to predict correctly, highlighting the superior coverage and robustness of EnzRetro.


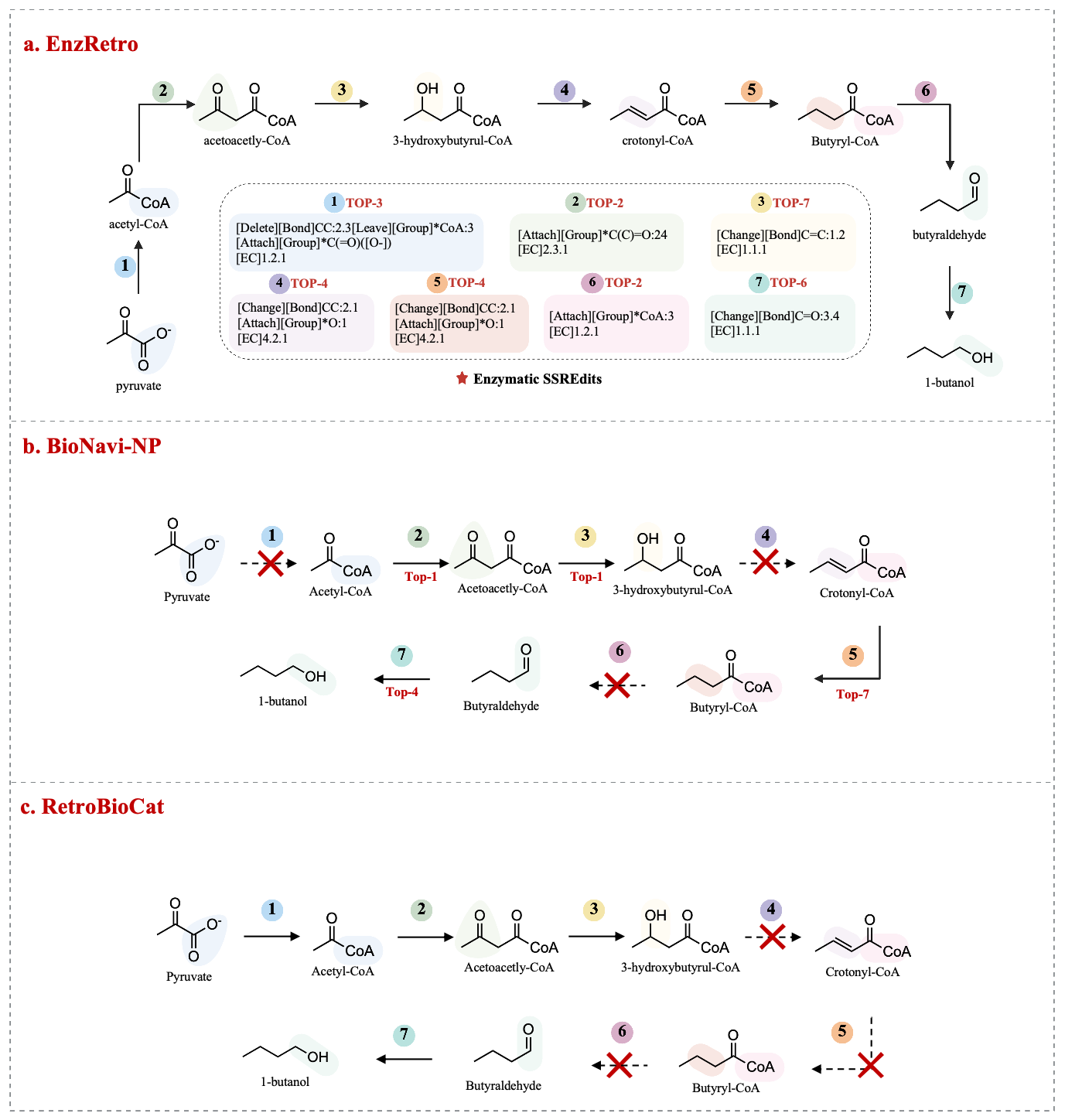


**Supplementary Figure 8. Comparative analysis of enzymatic retrosynthesis planning for the production of n-butanol.** The pathways are generated by (a) EnzRetro, (b) BioNavi-NP^[3]^ and (c) RetroBioCat^[4]^, respectively. EnzRetro successfully identifies a complete and feasible pathway. In contrast, dashed lines marked with a red cross denote reaction steps that the corresponding tools failed to predict correctly, highlighting the superior coverage and robustness of EnzRetro.


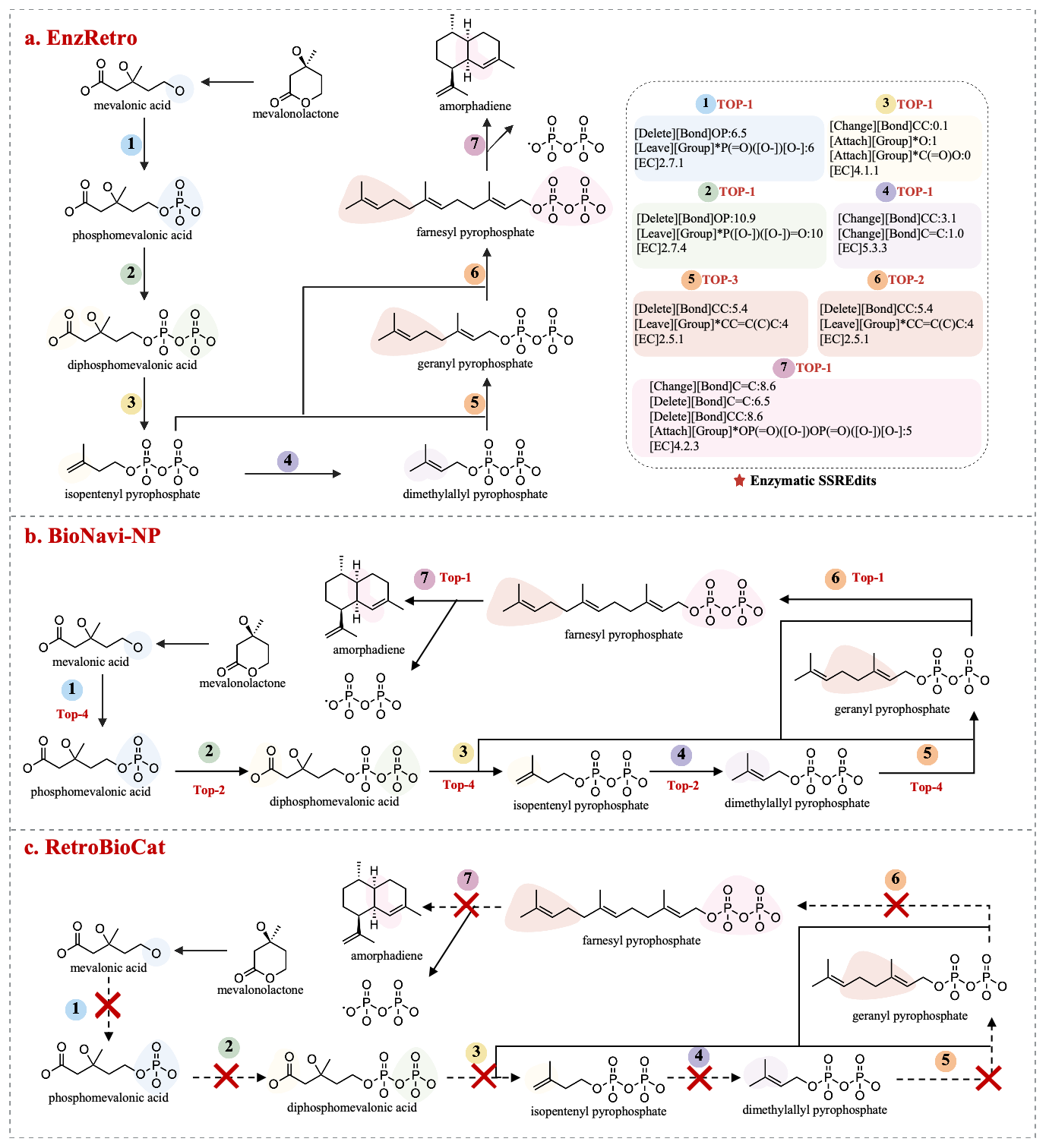


**Supplementary Figure 9. Comparative analysis of enzymatic retrosynthesis planning for the production of amorpha-4,11-diene.** The pathways are generated by (a) EnzRetro, (b) BioNavi-NP^[3]^ and (c) RetroBioCat^[4]^, respectively. EnzRetro successfully identifies a complete and feasible pathway. In contrast, dashed lines marked with a red cross denote reaction steps that the corresponding tools failed to predict correctly, highlighting the superior coverage and robustness of EnzRetro.

**Supplementary Tables**

**Supplementary Table I. Examples of enzymatic reactions and the corresponding enzymatic SSREdits for different EC numbers.**


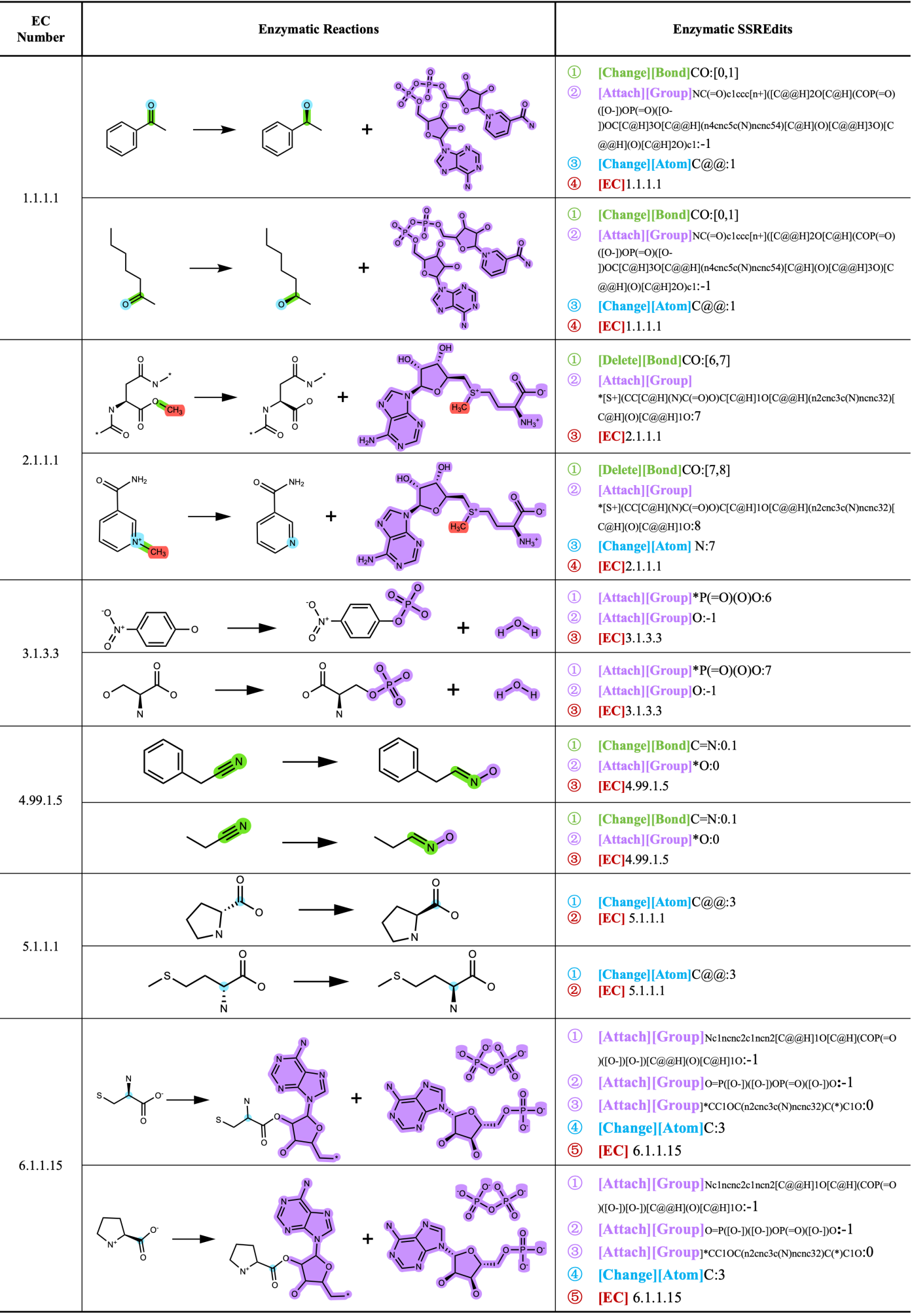
**Supplementary Table II. Action types of enzymatic SSREdits used for training EnzRetro for retro and forward synthesis.**

|  | **Retro** | **Forward** |
| --- | --- | --- |
| **Bond-level** | Delete Bond | Add Bond |
|  | Change Bond | Change Bond |
|  | Add Bond | Delete Bond |
| **Functional Group-level** | Attach Group | Leave Group |
|  | Leave Group | Attach Group |
| **Atom-level** | Change Atom | Change Atom |
| **Stop** | Terminate | Terminate |
| **EC Number** | EC | - |

***Note：***

1. **Delete Bond** deletes a bond between two atoms.
2. **Change Bond** changes bond type between two atoms.
3. **Add Bond** adds a new bond between two atoms.
4. **Attach Group** attaches a new functional group to the target molecule.
5. **Leave Group** leaves a functional group from the target molecule.
6. **Change Atom** represents the properties' changes of atoms, such as formal charge, chirality, or stereochemistry.
7. **Terminate** represents stopping generation and fixing H charges of the target molecule.
8. **EC** refers to the EC number for the enzymatic reaction.

*Note: The Enzyme Commission (EC) number is a hierarchical numerical system for classifying enzymes based on the chemical reactions they catalyze. It consists of four levels:

1. The first number (EC X.-.-.-) denotes the class, representing the general type of reaction (e.g., 1 for oxidoreductases, 2 for transferases).
2. The second number (EC X.X.-.-) denotes the subclass, providing more detail on the functional group acted upon or the type of bond formed/broken.
3. The third number (EC X.X.X.-) denotes the sub-subclass, which specifies the specific acceptor or donor group involved in the reaction, offering a fine-grained description of the reaction mechanism.
4. The fourth number (EC X.X.X.X) is the serial number, uniquely identifying a specific enzyme within its sub-subclass.

**Supplementary References**

[1] D. Probst, M. Manica, Y. G. Nana Teukam, A. Castrogiovanni, F. Paratore, T. Laino, *Nat Commun* **2022**, *13*, 964.

[2] P. D. Karp, R. Billington, R. Caspi, C. A. Fulcher, M. Latendresse, A. Kothari, I. M. Keseler, M. Krummenacker, P. E. Midford, Q. Ong, W. K. Ong, S. M. Paley, P. Subhraveti, *Brief Bioinform* **2019**, *20*, 1085.

[3] S. Zheng, T. Zeng, C. Li, B. Chen, C. W. Coley, Y. Yang, R. Wu, *Nat Commun* **2022**, *13*, 3342.

[4] W. Finnigan, L. J. Hepworth, S. L. Flitsch, N. J. Turner, *Nat Catal* **2021**, *4*, 98.
